# Author Correction: Reprogramming of regulatory network using expression uncovers sex-specific gene regulation in *Drosophila*

**DOI:** 10.1038/s41467-019-11418-z

**Published:** 2019-07-29

**Authors:** Yijie Wang, Dong-Yeon Cho, Hangnoh Lee, Justin Fear, Brian Oliver, Teresa M. Przytycka

**Affiliations:** 1 National Center of Biotechnology Information, National Library of Medicine, NIH, Bethesda, MD 20894 USA; 2 Laboratory of Cellular and Developmental Biology, National Institute of Diabetes and Digestive and Kidney Diseases, 50 South Drive, Bethesda, MD 20892 USA

**Keywords:** Gene regulatory networks, Network topology

Correction to: *Nature Communications* 10.1038/s41467-018-06382-z, published online 03 October 2018

The original version of this Article contained an error in Fig. 2, in which the performance of the MERLIN+P method shown in green in Fig. 2a, b, d, e was incorrectly reported. The correct version of Fig. 2 is

The original version of Fig. 2 is

This has been corrected in both the PDF and HTML versions of the Article.

