# Supplementary material for: Reprogramming of regulatory network using expression uncovers sex-specific gene regulation in Drosophila
Source: Nat Commun. 2018 Oct 3;9:4061. doi: 10.1038/s41467-018-06382-z (PMC6170494; doi:10.1038/s41467-018-06382-z)
Supplement: Supplementary file 1 — Supplementary Information [file 41467_2018_6382_MOESM1_ESM.pdf]

Reprogramming of regulatory network using expression uncovers sex-specific gene regulation in Drosophila

Yijie Wang, Dong-Yeon Cho, Hangnoh Lee, Justin Fear, Brian Oliver and Teresa M Przytycka

Supplementary Methods

NCA Model

Before describing the mathematical foundation of the NetREX method, we provide a brief overview of the traditional (static) network component analysis (NCA) method and its various implementations. Next we introduce the formula for the objective function in our NetREX method. Importantly, the objective function is non-convex and non-smooth because of the use of the ℓ_0 norm in our formulation. Rather than relaxing the problem by replacing the non-convex ℓ_0 norm with the convex ℓ_1 norm, we have directly solved the more challenging problem with the ℓ_0 norm by adapting the recently proposed Proximal Alternative Linearized Maximization (PALM) algorithm [1] to our original formulation.

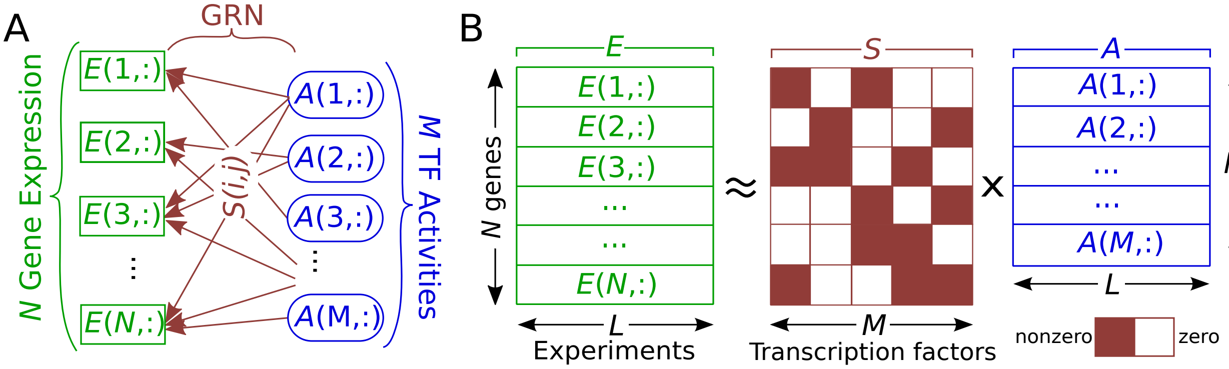

Supplementary Figure 1: The NCA model. (A) Graph representation of NCA. $E(i,:)$ is the expression of gene i over L experiments and $A(i,:)$ is the activity of TF i over the same L experiments. $S(i,j)$ is the control strength from TF j to gene i. (B) The algebraic formulation of NCA. E, S and A in (B) correspond to E, S and A in (A).

The main principle of NCA is to explain the expression of each gene as a linear combination of the activities of its regulating TFs, weighted by the strength of control they exert over that gene. The topology of the bipartite GRN is provided as a part of the input in NCA. Formally, let $E \in \mathbb{R}^{N\times L}$ be the matrix of expression data of N genes in L experiments. NCA is a special case of a more general problem which is to express E as

$$E = SA + \Gamma, \tag{1}$$

where $S \in \mathbb{R}^{N\times M}$ is the weighted adjacency matrix of the bipartite GRN $\mathcal{G}(TF, TG, \mathcal{S})$ such that the edges of $\mathcal{G}$ in the edge set $\mathcal{S}$ connect transcription factors in the M-element set TF to target genes from the N-element set TG. Specifically, for target gene i and transcription factor j, weight $S(i,j)$ defines the control strength that transcription factor j exerts on gene i. The ith row of $A \in \mathbb{R}^{M\times L}$, $A(i,:)$, represents the (hidden) TF activities of i over the set of experiments, and $\Gamma \in \mathbb{R}^{N\times M}$ represents the noise (Supplementary Fig. 1).

Many mathematical techniques, such as principle component analysis (PCA), independent component analysis (ICA), non-negative matrix factorization (NMF) [2] and sparse coding (SP) [3], can be used to determine the decomposition of E specified in (1) (for NMF, E needs to be normalized to a non-negative matrix). However, PCA and NMF [4] are unable to find a decomposition of E when $M > L$ (i.e. the number of TFs is larger than the number of experiments). Moreover, PCA and ICA hinge on assumptions of orthogonality and independence between the signals, which may

not hold for TF activities (rows of A). In addition, none of these techniques can utilize the prior knowledge of the GRN $\mathcal{G}$. In contrast, NCA [5, 6, 7, 8, 9, 10] can deal with the situation when $M > L$, makes no assumptions on TF activities, and is able to take full advantage of the prior knowledge of the GRN $\mathcal{G}$. Specifically, NCA aims to uncover the matrix A describing the hidden regulatory activities of TFs and the matrix S describing control strengths of each TF on target genes by assuming that the structure S_0 (unweighted adjacency matrix) of the underlying GRN $\mathcal{G}_0 = (TF, TG, \mathcal{S}_0)$ is known. That is, only the entries of S that correspond to edges in $\mathcal{S}_0$ can be non-zero (formally $\text{Support}(S) = \text{Support}(S_0)$, where $\text{Support}(S)$ denotes the support of S, i.e. the positions of its non-zero entries.). Thus NCA recovers the TF activities A and their control strengths S, with only the expression data E and the structure S_0 of $\mathcal{G}_0$ as inputs, by solving the following optimization problem.

$$
\begin{aligned}
&\min_{S,A} \frac{1}{2} \|E - SA\|_F^2 \\
&s.t.\ \text{Support}(S) = \text{Support}(S_0), \\
&\qquad \|S\|_\infty \leq a,\ \|A\|_\infty \leq b,
\end{aligned} \tag{2}
$$

where $\|S\|_\infty = \max_{i,j} |S(i,j)|$. The first constraint in the above formulation restricts the structure of the regulatory network $\mathcal{G}$, represented by matrix S, to be exactly the same as that of the input regulatory network $\mathcal{G}_0$. The rest of the constraints aim to ensure that the elements of A and S remain within the domain of biologically sensible values.

The first method [5] to solve (2) can only provide a unique solution if the following conditions are met: (i) the matrix S should have full-column rank; (ii) each column of S should have at least $M - 1$ zeros; (iii) the matrix A should have full row-rank. Under these conditions, S and A are estimated using an iterative two-step least-squares algorithm [5]. Tran et al. [6] expanded NCA by allowing the specification of the zero pattern of A as well as S. Galbraith et al. [7] modified the NCA method by revising the third criterion for NCA which cannot be tested before solving the problem. Chang et al. [8] treated NCA as an unconstrained optimization problem and employed singular value decomposition (SVD) to find a closed form solution for S without time-consuming iterations. Jacklin et al. [9] also proposed a non-iterative algorithm for NCA, resorting to convex optimization methods. All these methods are vulnerable to the presence of a small number of outliers in expression data. To deal with these outliers, Noor et al. [10] proposed ROBust Network Component Analysis (ROBNCA) where an additional sparse matrix was used for explicitly modeling the outliers.

The Formulation of NetREX

Aside from the numerous variants of NCA, the assumption that the GRN must be known in advance is a significant drawback to this method. NetREX relaxes this restriction under the assumption that a prior regulatory network that is not too far from the underlying true regulatory network is given. Therefore, it is possible to recover the underlying regulatory network by limited changes to the prior network. Note that this is a very reasonable assumption for many practical applications, as the prior network could come from a related organism, a related tissue, or even from the same organism but without sufficient data. Additionally, to guide network reconstruction, we assume that genes with highly correlated expression are likely to be regulated by the same TFs. The correlations between genes can be encoded in the gene correlation network $\mathcal{G}^E$, which is constructed based on gene expression data E. Thus, we remove the constraint that the structure of the network is fixed ($\text{Support}(S) = \text{Support}(S_0)$), but introduce a penalty term that limits the number of added and removed edges with respect to the prior network, along with terms encouraging consistent

treatment of co-expressed genes and network sparsity. The new optimization problem is defined as follows:

$$\min_{S,A} \frac{1}{2}\|E - SA\|_F^2 + \lambda\left(\|S_0\|_0 - \|S \odot S_0\|_0 + \left\|S \odot \bar{S}_0\right\|_0\right) + \kappa \mathrm{tr}(S^T L S) + \eta\|S\|_0 + \xi\|S\|_F^2 + \mu\|A\|_F^2$$
$$s.t.\ \ \|S\|_\infty \le a,\ \|A\|_\infty \le b. \tag{3}$$

where $\lambda, \kappa, \eta, \xi$, and μ are the parameters controlling the strength of the corresponding terms. We devote the rest of this subsection to explaining the roles of the added terms.

The term controlled by λ restricts the number of edge changes. Here $\bar{S}_0$ is the adjacency matrix of the complement graph of $\mathcal{G}_0$ and therefore $\bar{S}_0 + S_0 = \mathbf{1}_{N\times M}$. $\|X\|_0$ is the ℓ_0 norm that computes the number of non-zero entries in X. $\odot$ is the Hadamard product. We note that $\|S_0\|_0 - \|S \odot S_0\|_0$ denotes the exact number of regulations removed from $\mathcal{G}_0$ and $\left\|S \odot \bar{S}_0\right\|_0$ is the number of regulations added to the prior network $\mathcal{G}_0$. λ controls the change in topology of the regulatory network. A larger λ indicates that only a small number of edges can be added and removed, thereby controlling how far our predicted network $\mathcal{G}$ is from the prior network $\mathcal{G}_0$.

The term controlled by κ (the graph embedding term [11]) encourages $S(i,k)$ and $S(j,k)$ to have similar control strength if genes i and j are correlated. Here we provide derivations demonstrating that

$$\begin{aligned}
&\frac{1}{2}\sum_{i,j}\sum_{k} W(i,j)\left(S(i,k) - S(j,k)\right)^2 \\
&= \frac{1}{2}\sum_{i,j} W(i,j)\|S(i,:) - S(j,:)\|_2^2 \\
&= \frac{1}{2}\left(\sum_{i,j} W(i,j)S(i,:)S(i,:)^T + \sum_{i,j} W(i,j)S(j,:)S(j,:)^T - 2\sum_{i,j} W(i,j)S(i,:)S(j,:)^T\right) \\
&= \frac{1}{2}\left(\sum_{i} D(i,i)S(i,:)S(i,:)^T + \sum_{j} D(j,j)S(j,:)S(j,:)^T - 2\sum_{i,j} W(i,j)S(i,:)S(j,:)^T\right) \\
&= \frac{1}{2}\left(2\mathrm{tr}(S^T D S) - 2\mathrm{tr}(S^T W S)\right) \\
&= \mathrm{tr}(S^T L S),
\end{aligned} \tag{4}$$

where tr() is the trace of a matrix, and W and L are the adjacency matrix and the Laplacian matrix of the correlation network $\mathcal{G}^E$, respectively.

The term controlled by parameter η in (3) encourages sparsity of the final network (recall that the ℓ_0 norm computes the number of non-zero elements). However, we note that there may exist correlations between TF activities (rows of A), implying relationships between TFs that enforcing sparsity might weaken. This means that, for a gene, only one TF can be selected from a group of TFs whose activities are highly correlated, even though all TFs in the group regulate the gene. Therefore, we have an additional term (controlled by parameter ξ) that uses the Frobenius norm to encourage all regulating TFs to have non-zero values in S. For the reader familiar with the elastic net model, we point out that $\eta\|S\|_0 + \xi\|S\|_F^2$ is analogous to the ℓ_1 elastic net [12], and we can refer to it as the ℓ_0 elastic net.

Finally, the term controlled by the variable μ enforces smoothness of activities in A by not allowing many of its elements to reach the limit $\{-b, b\}$.

After some linear algebra, we obtain our final formulation as follows:

$$\begin{aligned}&\min_{S,A} \frac{1}{2}\|E - SA\|_F^2 + (\eta - \lambda)\|S \odot S_0\|_0 + (\eta + \lambda)\left\|S \odot \bar{S}_0\right\|_0 + \kappa \mathrm{tr}(S^T LS) + \xi \|S\|_F^2 + \mu \|A\|_F^2 \\ &s.t.\ \|S\|_\infty \leq a,\ \|A\|_\infty \leq b.\end{aligned} \tag{5}$$

We require $\eta - \lambda \geq 0$, otherwise the above formulation would preserve all regulations in $\mathcal{G}_0$.

Optimization Behind the NetREX Algorithm

Our algorithm to solve (5) relies on the recently proposed proximal alternative linearized maximization (PALM) [1] algorithm. The PALM method can solve a general optimization problem formulated as

$$\min : H(S, A) = F(S, A) + \Phi(S) + \Psi(A) \text{ over } S \in \Upsilon, A \in \Omega, \tag{6}$$

where $F(S, A)$ has to be smooth but $\Phi(S)$ and $\Psi(A)$ do not need to have the convexity and smoothness properties. Υ and Ω are constraint sets for S and A, respectively. The PALM algorithm alternatively applies a technique known as the proximal forward-backward scheme to both S and A. Specifically, at iteration k, the proximal forward-backward mappings of $\Phi(S)$ and $\Psi(A)$ on $S \in \Upsilon$ and $A \in \Omega$ for given S^k and A^k are the solutions for the following sub-problems, respectively,

$$S^{k+1} \in \arg\min_{S\in\Upsilon} \left\{ \left\langle S - S^k,\ \nabla_S F(S^k, A^k) \right\rangle + \frac{c^k}{2} \left\| S - S^k \right\|_F^2 + \Phi(S) \right\}; \tag{7a}$$

$$A^{k+1} \in \arg\min_{A\in\Omega} \left\{ \left\langle A - A^k,\ \nabla_A F(S^{k+1}, A^k) \right\rangle + \frac{d^k}{2} \left\| A - A^k \right\|_F^2 + \Psi(A) \right\}, \tag{7b}$$

where $\langle X, Y \rangle = \mathrm{tr}(X^T Y)$, c^k and d^k are positive real numbers and $\nabla_S F(S^k, A^k)$ is the derivative of $F(S, A^k)$ with respect to S at point S^k for fixed A^k and $\nabla_A F(S^{k+1}, A^k)$ is the derivative of $F(S^{k+1}, A)$ with respect to A at point A^k for fixed S^{k+1}. It has been proven that the sequence $\{(S^k, A^k)\}_{k\in\mathbb{N}}$ generated by PALM converges to a critical point when it is bounded [1].

Casting our optimization problem (5) into the PALM algorithm framework introduced in (6), we have $F(S, A) := \frac{1}{2}\|E - SA\|_F^2 + \kappa \mathrm{tr}(S^T LS)$, $\Psi(A) := \mu \|A\|_F^2$ and $\Phi(S) := (\eta + \lambda)\left\|\bar{S}_0 \odot S\right\|_0 + (\eta - \lambda)\|S_0 \odot S\|_0 + \xi \|S\|_F^2$. The constraint sets Υ and Ω are, respectively, $\Upsilon = \{S \mid \|S\|_\infty \leq a\}$ and $\Omega = \{A \mid \|A\|_\infty \leq b\}$. We note that $F(S, A)$, $\Psi(A)$, and $\Phi(S)$ satisfy the requirements of the PALM algorithm. Namely, $F(S, A)$ is smooth, $\Psi(A)$ is convex and smooth but, as allowed in the PALM approach, $\Phi(S)$ is non-convex and non-smooth. Hence, we can apply the PALM algorithm to our problem as long as we can efficiently solve the proximal forward-backward mappings for our specific $\Phi(S)$ and $\Psi(A)$. Proving that we can actually do this is mathematically the most challenging component in the development of the method. Due to the technicality of the derivations we leave most of them to the supplement and in what follows we only point to the most critical components of the argument.

It is easy to confirm that the NetREX problem (5) can be solved by alternatively applying the following proximal forward-backward mappings (8a) and (8b), which are derived from (7a) and (7b) by casting our specific $F(S, A)$, $\Phi(S)$, $\Psi(A)$, Υ and Ω and some linear algebra:

$$S^{k+1} \in \arg\min_{\|S\|_\infty \leq a} \left\{ \Phi(S) + \frac{c^k}{2} \left\| S - U^k \right\|_F^2 \right\}; \tag{8a}$$

$$A^{k+1} \in \arg\min_{\|A\|_\infty \leq b} \left\{ \Psi(A) + \frac{d^k}{2} \left\| A - V^k \right\|_F^2 \right\}, \tag{8b}$$

where

$$U^k = S^k - \frac{1}{c^k}\nabla_S F(S^k, A^k) \text{ and } V^k = A^k - \frac{1}{d^k}\nabla_A F(S^{k+1}, A^k). \quad (9)$$

The derivatives $\nabla_S F(S^k, A^k)$ and $\nabla_A F(S^{k+1}, A^k)$ can be computed by

$$\nabla_S F(S^k, A^k) = (S^k A^k - E)(A^k)^T + 2\kappa L S^k \text{ and } \nabla_A F(S^{k+1}, A^k) = (S^{k+1})^T(S^{k+1}A^k - E), \quad (10)$$

which are Lipschitz continuous with $L(A^k) = \left\|A^k(A^k)^T\right\|_F + 2\kappa\left\|L\right\|_F$ and $L(S^{k+1}) = \left\|(S^{k+1})^T S^{k+1}\right\|_F$ as Lipschitz constants, respectively. As suggested by [1], we set $c^k = \max\left\{v,\ L(A^k)\right\},\ v > 0$ and $d^k = \left\{v,\ L(S^{k+1})\right\},\ v > 0$ to make sure the formulas in (9) are well defined.

The closed form solution of the proximal forward-backward mapping (8a) can be obtained based on Proposition 1, the Proximal Mapping of the ℓ_0 Elastic Net Under $\|\|_\infty$ Constraint Proposition, and its corollary (Corollary 1). The proposition and corollary, along with their proofs, can be found in the following. We emphasize that Proposition 1 provides the closed form solution for the proximal mapping of the ℓ_0 elastic net under $\|\|_\infty$ constraint and thus it has broader applications to diverse feature selection approaches [13, 14].

Proposition 1 (**Proximal Mapping of the ℓ_0 Elastic Net Under the $\|\|_\infty$ Constraint**). *For a given $Y \in \mathbb{R}^{m\times n}$, the proximal mapping of the ℓ_0 elastic net under the $\|\|_\infty$ norm constraint is*

$$\arg\min_{\|X\|_\infty \le C}\left\{\|Y - X\|_F^2 + b\|X\|_F^2 + c^2\|X\|_0\right\} = T_{\frac{c}{\sqrt{b+1}}}\left(\mathbb{P}_{\|\cdot\|_\infty \le C}\left(\frac{Y}{b+1}\right)\right), \quad (11)$$

where the projection operator $\mathbb{P}_{\|\cdot\|_\infty \le C}(\cdot)$ is defined as

$$\mathbb{P}_{\|\cdot\|_\infty \le C}(Y) := \arg\min\left\{\|Y - X\|_F^2 : \|X\|_\infty \le C\right\} = \operatorname{sign}(Y)\odot\max\left\{|Y|, C\right\}, \quad (12)$$

such that the $||$, $\operatorname{sign}()$ and $\max\{\}$ operations are taken component-wise, and the hard-thresholding operator $T_c(\cdot)$ is defined as

$$T_c(Y) := \arg\min_X\left\{\|Y - X\|_F^2 + c^2\|X\|_0\right\}, \quad (13)$$

where $Y \in \mathbb{R}^{m\times n}$ is any given matrix and $T_c : \mathbb{R}^{m\times n} \to \mathbb{R}^{m\times n}$ is a component-wise mapping that can be explicitly written

$$(T_c(Y))(i,j) = \begin{cases} Y(i,j), & \text{if } |Y(i,j)| > c; \\ \{0, c\}, & \text{if } |Y(i,j)| = c; \\ 0, & o.w.. \end{cases} \quad (14)$$

Proof.

$$\begin{aligned} &\arg\min_{\|X\|_\infty \le C}\left\{\|Y - X\|_F^2 + b\|X\|_F^2 + c^2\|X\|_0\right\} \\ =&\arg\min_{\|X\|_\infty \le C}\left\{(b+1)\left\|\frac{Y}{b+1} - X\right\|_F^2 + c^2\|X\|_0\right\} \\ =&\arg\min_{\|X\|_\infty \le C}\left\{\left\|\frac{Y}{b+1} - X\right\|_F^2 + (\frac{c}{\sqrt{b+1}})^2\|X\|_0\right\} \\ =&T_{\frac{c}{\sqrt{b+1}}}\left(\mathbb{P}_{\|\cdot\|_\infty \le C}\left(\frac{Y}{b+1}\right)\right). \end{aligned} \quad (15)$$

The derivation of the last equality is based on Lemma 1. □

Lemma 1. *Let $U \in \mathbb{R}^{m\times n}$, then*

$$\arg\min\left\{\|U-X\|_F^2 + c^2\|X\|_0 : \|X\|_\infty \le C\right\} = T_c\left(\mathbb{P}_{\|\cdot\|_\infty\le C}(U)\right). \tag{16}$$

Proof. For a given $U \in \mathbb{R}^{m\times n}$, let us introduce the following notations

$$\|X\|_+^2 = \sum_{(i,j)\in\mathcal{I}^+} X(i,j)^2 \text{ and } \|X\|_-^2 = \sum_{(i,j)\in\mathcal{I}^-} X(i,j)^2, \tag{17}$$

where

$$\mathcal{I}^+ = \{(i,j)\in\{1,...,m\}\times\{1,...,n\} : |U(i,j)| \le C\} \tag{18}$$

and

$$\mathcal{I}^- = \{(i,j)\in\{1,...,m\}\times\{1,...,n\} : |U(i,j)| > C\} \tag{19}$$

The following observations hold

$$\begin{aligned} &(i)\ \|X\|_F^2 = \|X\|_+^2 + \|X\|_-^2 \\ &(ii)\ \|X-U\|_+^2 + \|X-C\|_-^2 = \left\|X - \mathbb{P}_{\|\cdot\|_\infty\le C}(U)\right\|_F^2 \\ &(iii)\ \|X-C\|_-^2 = 0 \Leftrightarrow X(i,j) = C\ \forall (i,j)\in\mathcal{I}^- \end{aligned} \tag{20}$$

where the second observation follows from observation (i) and the fact that $\left(\mathbb{P}_{\|\cdot\|_\infty\le C}(U)\right)(i,j) = U(i,j)$ for $(i,j)\in\mathcal{I}^+$ and $\left(\mathbb{P}_{\|\cdot\|_\infty\le C}(U)\right)(i,j) = C$ for $(i,j)\in\mathcal{I}^-$.

Based on the above facts, we have that $\bar{X} \in \text{prox}_{\|\cdot\|_0}^{\|\cdot\|_\infty\le C}(U,c)$ if and only if

$$\bar{X} \in \arg\min\left\{\|U-X\|_F^2 + c^2\|X\|_0 : \|X\|_\infty \le C\right\} \tag{21a}$$

$$= \arg\min\left\{\|U-X\|_+^2 + \|U-X\|_-^2 + c^2\|X\|_0 : \|X\|_\infty \le C\right\} \tag{21b}$$

$$= \arg\min\left\{\|U-X\|_+^2 + c^2\|X\|_0 : X(i,j) = C\ \forall(i,j)\in\mathcal{I}^-,\ \|X\|_\infty \le C\right\}, \tag{21c}$$

where the last equality follows from the fact that the solution of (21c) is also the solution of (21b), while the converse follows by a simple contradiction argument. Furthermore, one finds that the constraint $\|X\|_\infty \le C$ may be removed without affecting the optimal solution of the problem. Therefore, applying observations (ii) and (iii), we obtain

$$\begin{aligned} \bar{X} &\in \arg\min\left\{\|U-X\|_+^2 + c^2\|X\|_0 : \|X-C\|_-^2 = 0\right\} \\ &= \arg\min\left\{\|U-X\|_+^2 + \|X-C\|_-^2 + c^2\|X\|_0\right\} \\ &= \arg\min\left\{\left\|X - \mathbb{P}_{\|\cdot\|_\infty\le C}(U)\right\|_F^2 + c^2\|X\|_0\right\} = T_c\left(\mathbb{P}_{\|\cdot\|_\infty\le C}(U)\right), \end{aligned} \tag{22}$$

where the last equality is the definition of T_c in Eq. (15). □

Corollary 1. *For a given $U \in \mathbb{R}^{m\times n}$, the proximal mapping of $\Phi(S) = \alpha\left\|\bar{S}_0 \odot S\right\|_0 + \beta\|S_0 \odot S\|_0 + \gamma\|S\|_F^2$ on $\|S\|_\infty \le C$ is*

$$\begin{aligned} \text{prox}_{\|\cdot\|_\infty\le C}(U,\alpha,\beta,\gamma) &\in \arg\min_{\|S\|_\infty\le C}\left\{\alpha\left\|\bar{S}_0\odot S\right\|_0 + \beta\|S_0\odot S\|_0 + \gamma\|S\|_F^2 + \|U-S\|_F^2\right\} \\ &= T_{\sqrt{\frac{\beta}{\gamma+1}}}\left(\mathbb{P}_{\|\cdot\|_\infty\le C}\left(\frac{\mathbb{U}}{\gamma+1}\right)\right) + T_{\sqrt{\frac{\alpha}{\gamma+1}}}\left(\mathbb{P}_{\|\cdot\|_\infty\le C}\left(\frac{\bar{\mathbb{U}}}{\gamma+1}\right)\right), \end{aligned} \tag{23}$$

where $\mathbb{U} = S_0 \odot U$ and $\bar{\mathbb{U}} = \bar{S}_0 \odot U$.

Proof. We know that U can be decomposed into $U = \mathbf{1} \odot U = (S + \bar{S}_0) \odot U = S \odot U + \bar{S}_0 \odot U = \mathbb{U} + \bar{\mathbb{U}}$. Similarly, $S = S \odot S + \bar{S}_0 \odot S = \mathbb{S} + \bar{\mathbb{S}}$. Applying these decompositions in Eq. (23), we can decompose the proximal mapping into two parts.

$$\begin{aligned}&\arg\min_{\|S\|_\infty \le C} \left\{ \alpha \left\| \bar{S}_0 \odot S \right\|_0 + \beta \left\| S_0 \odot S \right\|_0 + \gamma \left\| S \right\|_F^2 + \left\| U - S \right\|_F^2 \right\} \\ &= \arg\min_{\|\mathbb{S}\|_\infty \le C} \left\{ \beta \left\| \mathbb{S} \right\|_0 + \gamma \left\| \mathbb{S} \right\|_F^2 + \left\| \mathbb{U} - \mathbb{S} \right\|_F^2 \right\} + \arg\min_{\|\bar{\mathbb{S}}\|_\infty \le C} \left\{ \alpha \left\| \bar{\mathbb{S}} \right\|_0 + \gamma \left\| \bar{\mathbb{S}} \right\|_F^2 + \left\| \bar{\mathbb{U}} - \bar{\mathbb{S}} \right\|_F^2 \right\}.\end{aligned} \tag{24}$$

Based on the Proximal Mapping of the ℓ_0 Elastic Net Proposition 1, we know

$$\arg\min_{\|\mathbb{S}\|_\infty \le a} \left\{ \beta \left\| \mathbb{S} \right\|_0 + \gamma \left\| \mathbb{S} \right\|_F^2 + \left\| \mathbb{U} - \mathbb{S} \right\|_F^2 \right\} = T_{\sqrt{\frac{\beta}{\gamma+1}}} \left(\mathbb{P}_{\|\cdot\|_\infty \le C} \left(\frac{\mathbb{U}}{\gamma + 1} \right) \right). \tag{25}$$

Similarly,

$$\arg\min_{\|\bar{\mathbb{S}}\|_\infty \le a} \left\{ \alpha \left\| \bar{\mathbb{S}} \right\|_0 + \gamma \left\| \bar{\mathbb{S}} \right\|_F^2 + \left\| \bar{\mathbb{U}} - \bar{\mathbb{S}} \right\|_F^2 \right\} = T_{\sqrt{\frac{\alpha}{\gamma+1}}} \left(\mathbb{P}_{\|\cdot\|_\infty \le C} \left(\frac{\bar{\mathbb{U}}}{\gamma + 1} \right) \right). \tag{26}$$

Combining Eq. (25) and Eq. (26) proves the proposition.

$$\begin{aligned}&\arg\min_{\|S\|_\infty \le C} \left\{ \alpha \left\| \bar{\mathbb{S}}_0 \odot S \right\|_0 + \beta \left\| \mathbb{S}_0 \odot S \right\|_0 + \gamma \left\| S \right\|_F^2 + \left\| U - S \right\|_F^2 \right\} \\ &= T_{\sqrt{\frac{\beta}{\gamma+1}}} \left(\mathbb{P}_{\|\cdot\|_\infty \le C} \left(\frac{\mathbb{U}}{\gamma + 1} \right) \right) + T_{\sqrt{\frac{\alpha}{\gamma+1}}} \left(\mathbb{P}_{\|\cdot\|_\infty \le C} \left(\frac{\bar{\mathbb{U}}}{\gamma + 1} \right) \right).\end{aligned} \tag{27}$$

□

With the help of Proposition 1 and Corollary 1, (8a) can be efficiently computed by

$$S^{k+1} \in \mathrm{prox}_{\|\cdot\|_\infty \le a} \left(U^k, \frac{2(\eta + \lambda)}{c^k}, \frac{2(\eta - \lambda)}{c^k}, \frac{2\xi}{c^k} \right), \tag{28a}$$

And (8b) can be computed by

$$A^{k+1} = \mathbb{P}_{\|\cdot\|_\infty \le b} \left(\frac{1}{1 + \frac{2\mu}{d^k}} V^k \right). \tag{28b}$$

The definitions of $\mathrm{prox}_{\|\cdot\|_\infty \le a}(\cdot)$ and $\mathbb{P}_{\|\cdot\|_\infty \le b}(\cdot)$ can be found in Corollary 1 and Proposition 1, respectively. The derivations of (28a) and (28b) can be found in the following.

The derivation for (28a) using Corollary 1 is shown below.

$$\begin{aligned}S^{k+1} &\in \arg\min_{\|S\|_\infty \le a} \left\{ \Phi(S) + \frac{c^k}{2} \left\| S - U^k \right\|_F^2 \right\} \\ &= \arg\min_{\|S\|_\infty \le a} \left\{ \frac{2(\eta + \lambda)}{c^k} \left\| \bar{\mathbb{S}}_0 \odot S \right\|_0 + \frac{2(\eta - \lambda)}{c^k} \left\| \mathbb{S}_0 \odot S \right\|_0 + \frac{2\xi}{c^k} \left\| S \right\|_F^2 + \left\| S - U^k \right\|_F^2 \right\} \\ &= \mathrm{prox}_{\|\cdot\|_\infty \le a} \left(U^k, \frac{2(\eta + \lambda)}{c^k}, \frac{2(\eta - \lambda)}{c^k}, \frac{2\xi}{c^k} \right).\end{aligned} \tag{29}$$

The derivation for (28b) is shown below.

$$
\begin{aligned}
A^{k+1} &= \arg \min_{\|A\|_\infty \le b} \left\{ \Psi\left(A\right) + \frac{d^k}{2} \left\| A - V^k \right\|_F^2 \right\} \\
&= \arg \min_{\|A\|_\infty \le b} \left\{ \frac{2\mu}{d^k} \left\| A \right\|_F^2 + \left\| A - V^k \right\|_F^2 \right\} \\
&= \mathbb{P}_{\|\cdot\|_\infty \le b} \left(\frac{1}{1 + \frac{2\mu}{d^k}} V^k \right).
\end{aligned}
\tag{30}
$$

We now have all the ingredients for our NetREX algorithm. Hence, we describe the NetREX algorithm in Algorithm 1. We note that the constraints for both S and A ($\|S\|_\infty \le a$ and $\|A\|_\infty \le b$) make sure that the sequence $\left\{(S^k, A^k)\right\}_{k\in\mathbb{N}}$ is bounded. Thus we state that the sequence produced by the NetREX algorithm converges to a critical point of the optimization problem (5), which is described in Proposition 2.

Algorithm 1: The NetREX algorithm.

Input : S_0, E, L, η, λ, κ, ξ, $v > 0$ and K;
Output: S and A.

begin
- (S^0, A^0) =Initialization(S_0). // Algorithm 2.
- **for** $k = 0, 1, 2, ..., K$ **do**
  - $c^k = \max\left\{v,\ L(A^k)\right\}$.
  - $U^k = S^k - \frac{1}{c^k}\left(S^k A^k (A^k)^T + 2\kappa L S^k - E(A^k)^T\right)$. // put (10) into (9).
  - $S^{k+1} \in \text{prox}_{\|\cdot\|_\infty \le a}\left(U^k, \frac{2\eta}{c^k}, \frac{2(\eta-\lambda)}{c^k}, \frac{2\xi}{c^k}\right)$. //as shown in (28a).
  - $d^k = \left\{v,\ L(S^{k+1})\right\}$.
  - $V^k = A^k - \frac{1}{d^k}\left((S^{k+1})^T (S^{k+1}) A^k - (S^{k+1})^T E\right)$. // put (10) into (9).
  - $A^{k+1} = \mathbb{P}_{\|\cdot\|_\infty \le b}\left(\frac{1}{1 + \frac{2\mu}{d^k}} V^k\right)$. //as shown in (28b).
- **end**
- $S = S^K$ and $A = A^K$

end

To ensure that the starting point is consistent with the prior network, (S^0, A^0) must be inferred from our prior network $\mathcal{G}_0$. We thereby compute (S^0, A^0) by solving the following problem, which is obtained from dropping the constraints and disregarding the non-smooth regularization term $(\eta - \lambda)\left\|S \odot S_0\right\|_0 + (\eta + \lambda)\left\|S \odot \bar{S}_0\right\|$ of S in the original NetREX formulation.

$$
\min_{S,A} : J(S, A) = \frac{1}{2}\left\|E - SA\right\|_F^2 + \kappa \text{tr}(S^T L S) + \xi \left\|S\right\|_F^2 + \mu \left\|A\right\|_F^2 \tag{31}
$$

The problem (31) can be solved by the standard Gauss-Seidel scheme [15] that alternatively solves the multi-variable optimization problem with respect to one variable while fixing the rest of the variables. Specifically, we can fix $S = S_k^0$ and solve (31) with respect to the closed form of A shown in Line 4 of Algorithm 2. Then, we fix $A = A_k^0$ and solve (31) with respect to S, whose solution is the solution of the Sylvester equation $SA_k^0(A_k^0)^T + 2(\kappa L + \xi I)S = E(A_k^0)^T$ (derived by setting

$\nabla H(S, A_k^0) = 0$). The Sylvester equation is solved by the standard Bartels-Stewart algorithm. We alternatively run lines 4 and 5 K times. In the end, we project the solutions A_K^0 and S_K^0 onto the feasible space of Eq. (5) by the projection operator (12) shown in lines 7 and 8. Algorithm 2 elaborates the details of obtaining (S^0, A^0).

Algorithm 2: The initialization for NetREX

Function: Initialization(S_0);
Input : S_0;
Output : S^0 and A^0.

```
1 begin
2   S_0^0 = S_0.
3   for k = 0, 1, 2, ..., K do
4     A_k^0 = ((S_k^0)^T S_k^0 + μI)^{-1} (S_k^0)^T E.
5     S_{k+1}^0 := { Ŝ | Ŝ A_k^0 (A_k^0)^T + 2(κL + ξI) Ŝ = E (A_k^0)^T }.
6   end
7   A^0 = P_{||·||_∞ ≤ b}(A_K^0).
8   S^0 = P_{||·||_∞ ≤ a}(S_K^0).
9 end
```

Line 2: $S_0^0 = S_0$.
Line 4: $A_k^0 = \left((S_k^0)^T S_k^0 + \mu I\right)^{-1} (S_k^0)^T E$.
Line 5: $S_{k+1}^0 := \left\{\hat{S} | \hat{S} A_k^0 (A_k^0)^T + 2(\kappa L + \xi I)\hat{S} = E(A_k^0)^T\right\}$.
Line 7: $A^0 = \mathbb{P}_{\|\cdot\|_\infty \le b}(A_K^0)$.
Line 8: $S^0 = \mathbb{P}_{\|\cdot\|_\infty \le a}\left(S_K^0\right)$.

Proposition 2 (**Convergnece Proposition**). *Let $\left\{(S^k, A^k)\right\}_{k\in\mathbb{N}}$ be a sequence generated by the NetREX algorithm. Then,*
(i) *The sequence $\left\{(S^k, A^k)\right\}_{k\in\mathbb{N}}$ has finite length, that is*

$$\sum_{k=1}^{\infty} \left\|S^{k+1} - S^k\right\|_F + \left\|A^{k+1} - A^k\right\|_F < \infty. \tag{32}$$

(ii) *The sequence $\left\{(S^k, A^k)\right\}_{k\in\mathbb{N}}$ converges to a critical point (S^*, A^*) of the NetREX problem.*

Proof. We apply Theorem 3.1 in [1] to guarantee that the sequence generated by NetREX is globally convergent to the critical points of (5). □

An Alternative Formulation

There is an alternative formulation for (5):

$$\begin{aligned} \min_{S,A} \ & \frac{1}{2}\|E - SA\|_F^2 + \kappa \mathrm{tr}(S^T L S) + \xi \|S\|_F^2 + \mu \|A\|_F^2 \\ s.t. \ & \|S \odot S_0\|_0 \le \mathcal{U}, \ \left\|S \odot \bar{S}_0\right\|_0 \le \mathcal{V}, \\ & \|S\|_\infty \le a, \ \|A\|_\infty \le b. \end{aligned} \tag{33}$$

The newly added constraints $\|S \odot S_0\|_0 \le \mathcal{U}$ and $\left\|S \odot \bar{S}_0\right\|_0 \le \mathcal{V}$ restrict the number of edges kept in the prior and added in the prior, respectively. The problem can be solved by using PALM (similar to the algorithm in Section 4 [1]). The details of the derivations are left to the audience.

The NetREX_NP and NetREX_ℓ_1 Algorithms

The NetREX_NP algorithm is the same as Algorithm 1 with $\lambda = 0$. The formulation of NetREX_NP is

$$\begin{aligned} &\min_{S,A} \frac{1}{2}\|E - SA\|_F^2 + \eta\|S\|_0 + \kappa \mathrm{tr}(S^T L S) + \xi\|S\|_F^2 + \mu\|A\|_F^2 \\ &s.t.\ \|S\|_\infty \le a,\ \|A\|_\infty \le b. \end{aligned} \tag{34}$$

This is similar to sparse coding [3] if we remove the graph embedding term.
The NetREX_ℓ_1 formulation is as follows

$$\begin{aligned} &\min_{S,A} \frac{1}{2}\|E - SA\|_F^2 + (\eta - \lambda)\|S \odot S_0\|_1 + (\eta + \lambda)\left\|S \odot \bar{S}_0\right\|_1 + \kappa \mathrm{tr}(S^T L S) + \xi\|S\|_F^2 + \mu\|A\|_F^2 \\ &s.t.\ \|S\|_\infty \le a,\ \|A\|_\infty \le b. \end{aligned} \tag{35}$$

To do a fair comparison we also solve using the PALM algorithm, which is analogous to Algorithm 1. The only difference is that in line 6 of Algorithm 1 we use the proximal mapping of the ℓ_1 elastic net given in [16] instead of a proximal mapping for the ℓ_0 elastic net.

Ranking Interactions and Bootstrapping

We rank every interaction $S(i,j)$ based on its impact on the modeling, computed by

$$B(i,j) = 1 - \frac{\left\|E(i,:) - \sum_{k \neq j} S(i,k)A(k,:)\right\|_F^2}{\|E(i,:) - S(i,:)A\|_F^2}, \tag{36}$$

where $B(i,j)$ is the confidence score. Then all interactions $S(i,j)\ \forall i,j$ can be ranked based on the corresponding confidence score $B(i,j)$. To further improve the inference against over-fitting and sampling errors, we use a bootstrapping strategy. We re-sample the expression data E with replacement and run NetREX on the new dataset. This procedure is repeated 5 times, and the resulting lists of interactions (B matrices) are rank combined to a final ranked list Λ as in [17].

Model Selection of NetREX

When we have a “gold standard” of partial GRNs, the parameters of NetREX can be selected based on the known “gold standard”.

If we reconstruct a GRN in a new context, where we do not have any prior knowledge, it is hard to select one set of optimal parameters for NetREX. To avoid that, we can apply a grain-grid search on the parameter space. Once we pick a set of parameters in the grain-grid, we then generate l sets of fine-grid parameters around it and use all those parameters to get several ranking matrices $\Lambda_i,\ i = 1, ..., l$. Again we use the ranking scheme [17] to do a consensus and get the final results from the Λs.

The PriorBoost Score

The assessment of the prior networks is based on two intuitions. First, the quality of the network can be estimated by the consistency between the structure of the network and the expression data. Such consistency can be computed by the following equation:

$$q(G) := \min_{S \in G, A} \|E - SA\|_F^2, \tag{37}$$

where G is the network we want to verify. $S \in G$ means that the non-zero pattern of S is conserved in the structure induced by G. Actually, (37) is the original formulation of NCA and $q(G)$ is the optimal objective function value after solving. Lower $q(G)$ implies better quality of the network G.

The first intuition is supported by the *E.coli* golden standard GRN (Supplementary Fig.2). As shown in Supplementary Fig.2, for random networks with a fixed number of total edges, the more experimentally verified edges that exist the lower the NCA fitting error achieved.

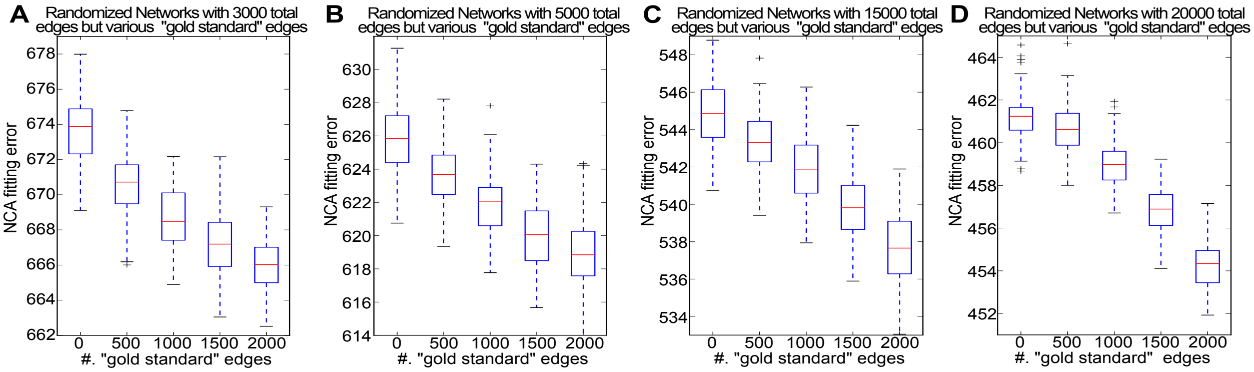

Supplementary Figure 2: NCA fitting error (37) vs. the number of experimentally verified regulatory edges. (A) Boxplots of NCA fitting error for random networks with 3000 edges, within which the number of "gold standard" edges varies from 0 to 2000. (B) Boxplots of NCA fitting error for random networks with 5000 edges, within which the number of "gold standard" edges various from 0 to 2000. (C) Boxplots of NCA fitting error for random networks with 10000 edges, within which the number of "gold standard" edges varies from 0 to 2000. (D) Boxplots of NCA fitting error for random networks with 20000 edges, within which the number of "gold standard" edges varies from 0 to 2000.

The second intuition we rely on is that, if a prior network is consistent with the given expression data, the network predicted by a prior-based method should be better than the network inferred by an expression-based method. The prior-based method we used here was NetREX, and the expression-based method we used was Genie3, which was the winner of the DREAM4 and DREAM5 challenges [18, 17].

Suppose we have a prior network G_0 and expression data E. G^* is the network predicted by NetREX via utilizing G_0 and $\bar{G}$ is the network predicted by Genie3 using E. G_c^* and $\bar{G}_c$ are networks obtained by keeping the top c edges in G^* and $\bar{G}$ based on the edge weights, respectively. Then, the PriorBoost score of the prior network G_0 can be estimated by

$$Q\left(G_0\right) := \frac{1}{|C|} \sum_{c \in C} q\left(\bar{G}_c\right) - q\left(G_c^*\right), \tag{38}$$

where C is a set of different cutoffs. Positive $Q\left(G_0\right)$ indicates that the network predicted by NetREX using G_0 is more consistent with the expression data E than the network predicted by Genie3, and negative $Q\left(G_0\right)$ indicates the opposite.

Supplementary Note 1. Results on Simulated Data

To validate our approach, we applied NetREX to the simulated data generated based on (1), the linear model. We first randomly generated the "gold standard" adjacency matrix S of the regulatory network $\mathcal{G}(TF, TG, \mathcal{S})$ and TF activities A. Then, the simulated expression data was generated as

$$E(i,j) = \sum_{p} S(i,p)A(p,j) + \Gamma(i,j), \tag{39}$$

where $\sum_p S(i,p)A(p,j)$ is the noiseless data arising from the known A and S matrices and the noise $\Gamma(i,j) \sim \mathbf{N}(0, \sigma^2)$ obeys a normal distribution with 0 mean and σ^2 variance. We assigned the prior network $\mathcal{G}_0$ the same number of edges as the "gold standard" network $\mathcal{G}$, but only θ percent of the edges in $\mathcal{G}_0$ are true edges. We can tune the difficulty of the network rewiring task by using different σ and θ. We set $S(i,p) \in \{0,1\}$, $A(p,j) \in [-1,1]$, and $\sigma \in [0,1]$. We further convert σ to signal to noise ratio (SNR) as shown in Supplementary Fig. 3.

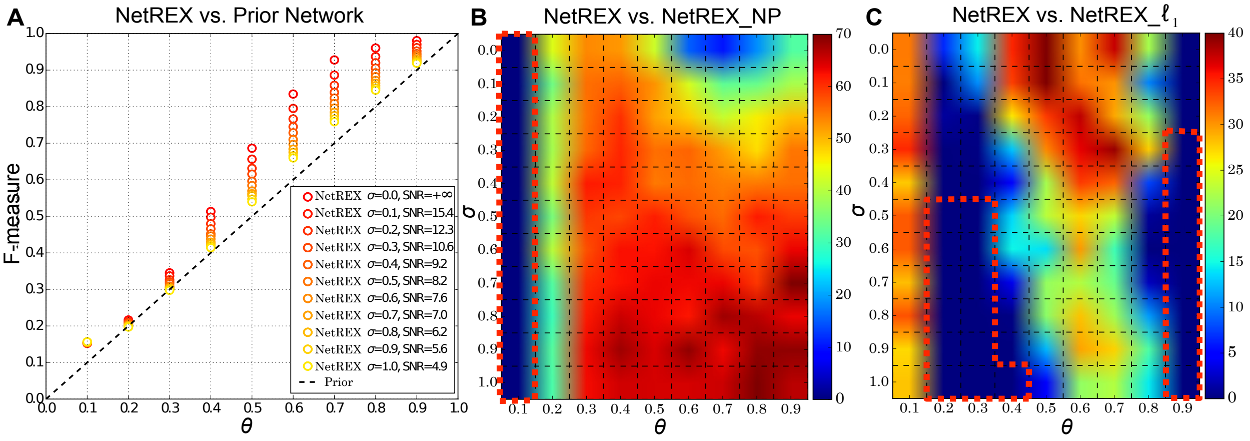

Supplementary Figure 3: (A) Comparison between F-measures of the networks predicted by NetREX and prior networks. The x-axis denotes percentage of true edges in the prior network and the black dashed line denotes F-measures of the prior networks. The circles are the average F-measures of the networks predicted by NetREX under different σ and θ over 50 random inputs. (B) Comparison between F-measures of the networks predicted by NetREX and NetREX_NP (Supplementary Method 5.). The color in each dashed block indicates the $-\log$ p-value for corresponding (σ, θ), where the p-values are obtained from a one-sided paired t-test between F-measures of the compared algorithms. The warmer the color is, the larger the F-measures of the networks predicted by NetREX over those of NetREX_NP. The red dashed line circles the (σ, θ) pairs where NetREX_NP achieves a larger F-measure at significance level 0.01. (C) Comparison between F-measures of the networks predicted by NetREX and NetREX_ℓ_1 (Supplementary Method 5.). The color coding is the same as in panel (B).

We evaluated the performance of the compared algorithms in terms of F-measure (Supplementary Note 5), which quantifies the overlap between the structures of the predicted network and the "gold standard" network. F-measure ranges from 0 to 1, where 1 indicates that the underlying $\mathcal{G}$ is fully recovered and 0 means the opposite. To avoid the effect of parameter selection, for each algorithm, under certain noise level (σ, θ), we first found its optimal parameters in terms of F-measure on one simulated dataset through grid search. Then we ran the algorithm on another 50 randomly generated simulated datasets under the same (σ, θ) using its optimal parameters. We can then

further test whether one method is statistically better than another method under a specific noise level by computing the p-value from a one-side paired t-test between all 50 paired F-measures. The detailed parameter settings are listed in Supplementary Note 6.

The comparisons between NetREX and these other methods are shown in Supplementary Fig. 3. Supplementary Fig. 3A shows the comparison between networks predicted by NetREX and the prior networks, in which we found that when the expression data is less noisy (σ is small) and the prior network is closer to the "gold standard" (θ is large), the network predicted by NetREX has a tendency to achieve higher F-measures than the prior networks. Additionally, we note that NetREX exhibits, by a larger margin, higher F-measures than the the prior networks after $\theta \geq 0.3$. However, for $\theta < 0.3$ the networks predicted by NetREX are only marginally better than the prior network, which implies that if we use random networks that do not have as much overlap with the "gold standard" as the prior networks, we cannot obtain promising results.

The effectiveness of the graph embedding term in (5) has been proved in [11], and the effectiveness of the ℓ_0 elastic net can be inferred from the results in [12]. In the following, we prove the function of the perturbation term controlled by λ and the superiority of using the ℓ_0 norm rather than the ℓ_1 norm via simulation data.

We compared NetREX with its two natural variants on the simulation data. The first variant is NetREX_NP (NetREX with No edge Perturbation term) that has the same formulation as NetREX but with $\lambda = 0$. The difference between NetREX and NetREX_NP is that NetREX penalizes the number of edges added and removed from the prior network but NetREX_NP does not. Here we should mention that NetREX_NP and sparse coding have similar formulations (Appendix 9). The other related algorithm in our comparison is NetREX_ℓ_1, which estimates the ℓ_0 norm in NetREX using the ℓ_1 norm. We note that substituting the ℓ_1 norm for the ℓ_0 norm makes the sub-problems convex and thus easier to solve. The implementation of these two algorithms is introduced in Appendix 9.

The comparison between NetREX and NetREX_NP is displayed in Supplementary Fig. 3B. We note that NetREX significantly outperforms NetREX_NP after $\theta > 0.1$. In Supplementary Fig. 3C, we observe that NetREX_ℓ_1 performs better in certain cases where the noise in the expression data is large (σ is large) because the ℓ_1 norm is robust to noise. However, for most noise levels, NetREX achieved significantly higher F-measures compared to NetREX_ℓ_1, demonstrating that the ℓ_0 norm is superior to the ℓ_1 norm for selecting sparse contributing components.

We conducted a "ONE-AT-A-TIME" [19] sensitivity analysis for all parameters as follows. For fixed (σ, θ), we first use grid search to find the optimal parameters as introduced in Supplementary Material Section E.2. Then, we tune one parameter from low to high while keeping all the rest of the parameters fixed at their optimum. Therefore, we can obtain the precision, recall, and F-measure according to the parameter we tune. Specifically, we set $\sigma = 0.2$ and $\theta = 0.8$ and for all parameters (including λ, η, κ, μ and ξ) we set them to the set $\{0.01,\ 0.1,\ 1,\ 10,\ 100,\ 1,000,\ 10,000\}$.

The box-plots of precision, recall, and F-measure while tuning one of the parameters are shown in Supplementary Fig. 4. We notice that setting η, μ and ξ extremely large leads to the trivial solutions $A = 0$ and $S = 0$. In this case, all three measures become 0. Setting κ extremely large makes S equal to the correlation network G^E and the corresponding measures are the ones for G^E. All parameters need to be carefully selected since they all are important for achieving promising results.

Supplementary Note 2. Results on Simulated Data with Non-random Errors

We generated the simulated data as follows. First, we randomly generated a GRN between M TFs and N genes (black edges in Supplementary Fig. 5 a). Then, we added a module of n genes

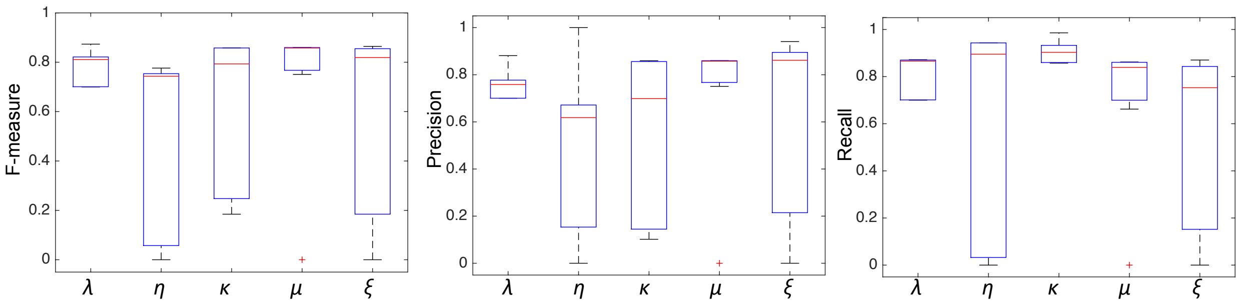

Supplementary Figure 4: Sensitivity of each parameter on F-measure, Precision and Recall.

and randomly selected m TFs to regulate the genes in the module. The regulatory interactions between these m TFs and genes in the module from a fully connected bipartite graph (green edges in Supplementary Fig. 5 a). Using so constructed true GRN, we generated expression data for $N+n$ genes using the linear model introduced in Supplementary Discussion equation (39) including the addition of expression noise.

To simulate the scenario where the prior is consistent with the true network in most cases except one truly differential module of genes, we randomly removed a subset of "true" edges connecting TFs to the genes in the module and randomly reattached them to wrong genes (red edges in Supplementary Fig. 5 a). Then we run NetREX using the so perturbed network as the prior and measured Recovery Accuracy [20] of the true edges leading to the module. We tested how the results depend on two factors: (i) the percentage of the rewired true edges (varied from 40% to 100%) and (ii) added expression noise (Supplementary Fig. 5 b). NetREX performed very well even in the case when all true edges leading to the module have been removed from the prior. The reason for this high performance can be attributed to the fact that TFs that regulate the module also regulate some genes outside the module allowing NetREX estimate their activities. Then the true edges between TFs and the modules could be recovered by utilizing these, since activities have the capacity to explain the expression of the genes in the module.

Supplementary Note 3. Results on *E.coli* Data

We used the "gold standard" *E.coli* GRN from DREAM5, which has 2066 interactions between 141 TFs and 4511 genes. We randomly constructed 10 simulated unweighted prior networks that have 2066 interactions but with different percentages of true edges within those 2066 interactions. We evaluated the performance of all prior-based methods, including MERLIN_P [21], Inferelator [22] and NetREX, by checking their ability to recover the "gold standard" *E.coli* GRN given prior networks with different percentages of true edges. The performance was quantified by AUROC and AUPR scores as shown in Supplementary Table 1 and Supplementary Table 2. Unlike the F-measure used in Section 9, which assesses the overlap between the structures of predicted networks and "gold standard" networks, AUROC and AUPR scores are two metrics that evaluate the ranking of the edges in the predicted networks. The selection of parameters for different methods is discussed in Supplementary Note 6. As shown in Supplementary Table 1, Supplementary Table 2, and Fig. 2 in the main text, NetREX significantly outperformed the competing algorithms and always achieved better AUROC and AUPR scores when the PriorBoost score was positive.

Another interesting observation is that the graph embedding term might slightly improve the AUROC and AUPR scores depending on the quality of the embedding co-expressed networks. Actually, if we have other reliable sources that contain information about the similarity of gene

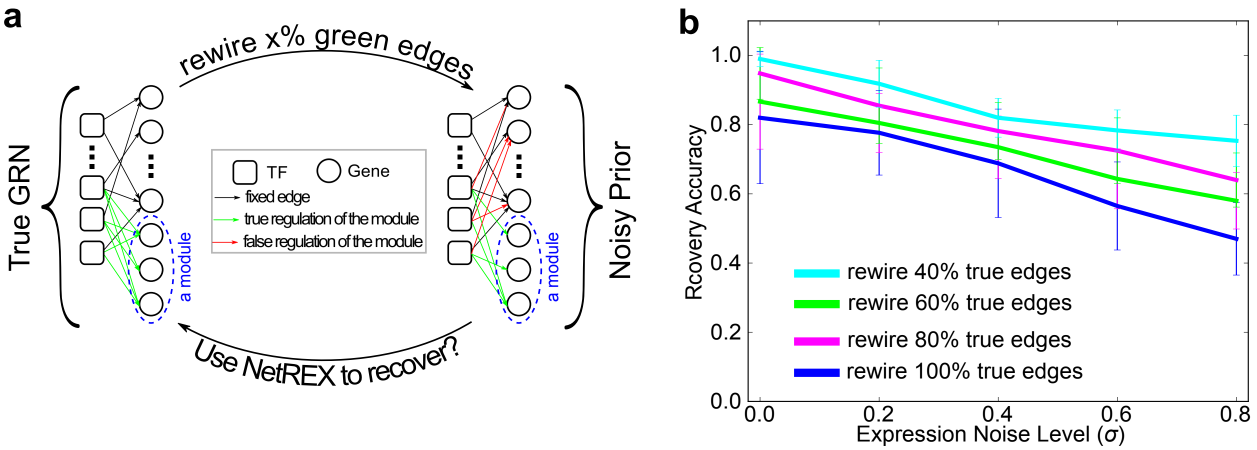

Supplementary Figure 5: Test NetREX performance under the malicious error model where the prior is consistent with the true network except one truly differential module of genes. (a) Construction of the test data. Rewire x% edges means that (1-x)% of true edges leading to the module (green edges) are kept (conserved) and the rest is connected to the wrong genes outside the module (red edges). (b) The performance of NetREX on recovery true edges in terms of Recovery Accuracy [20] under various percentage of rewired edges various level of added expression noise.

pairs, we can also embed them to make similar gene pairs co-regulated by similar TFs.

We further generated noisy unweighted prior networks in which we fixed the number of true edges (1033 for this experiment), but varied the ratio of true to false edges over a range of values (0:1, 1:0, 1:2, 1:5, 1:10). 0:1 means the prior network contains no "gold standard" edges but all wrong edges (0 "gold standard" edges and 1033 false edges). 1:0 means the prior network contains no false edges but all "gold standard" edges. Supplementary Table 3 and Supplementary Table 4 show the comparison in terms of AUROC and AUPR of all approaches on this data. Clearly, NetREX significantly outperforms Inferelator and MERLIN_P.

We extract novel TF-gene interactions with strong evidence in *E.coli* from RegulonDB 9.2 (version 09-08-2016) [23]. Other than the 2,066 existing interactions in DREAM5, we find 230 extra interactions not used in DREAM5. We use these 230 novel interactions to further validate the performance of the competing algorithms. From Supplementary Table 5 and Supplementary Table 6, which correspond to Fig.2b and Fig.2f in main text, we find that NetREX outperforms all competing algorithms at identifying these novel interactions.

Following the ideas proposed in [24], we modified two metrics, PPI score and GO score, to help evaluate the quality of the predicted networks when we do not have any "gold standard" information. The idea behind using PPI and GO scores is that gene pairs co-regulated by most of the same TFs should be functionally similar. Those gene pairs are more likely to have protein interactions and share similar GO terms comparing to random ones. PPI scores and GO scores are the statistical significance ($-\log(\text{p-value})$) obtained from a hypergeometric test.

To test this idea, we generated randomly simulated *E.coli* GRNs with different noise levels controlled by the percentage of true edges and the ratio of true to false edges. The PPI and GO scores were then computed for the simulated networks. We obverse that both the PPI and GO scores are consistent with the quality of the simulated networks. Namely, the less noisy the network, the larger the PPI and GO scores we observed. Based on this result, we were confident about the use of both scores for the adult fly networks in the following sections, where we do not have "gold standard" networks for validation.

Supplementary Table 1: The average and variance of AUROC scores of all competing prior-based algorithms. The best scores for prior networks with different percentage of true edges are in bold.

	Prior		MERLIN_P		Inferelator		NetREX($\kappa = 0$)		NetREX($\kappa = 1$)	
True edges %	AUROC	Var.	AUROC	Var.	AUROC	Var.	AUROC	Var.	AUROC	Var.
10%	0.5437	4.7E-11	0.5975	3.3E-6	0.5561	5.6E-5	**0.5668**	1.9E-5	**0.5668**	2.1E-5
20%	0.5944	2.6E-10	0.5947	4.8E-6	0.6132	4.5E-5	0.6367	2.0E-5	**0.6380**	2.5E-5
30%	0.6450	1.2E-10	0.5969	6.2E-6	0.6545	4.6E-5	**0.7020**	5.1E-6	0.7009	5.0E-6
40%	0.6958	7.1E-11	0.5975	2.0E-6	0.6874	5.2E-5	0.7579	1.8E-5	**0.7580**	1.6E-5
50%	0.7466	2.4E-10	0.5960	1.8E-6	0.7194	6.1E-5	**0.8069**	9.5E-6	**0.8069**	7.9E-6
60%	0.7971	9.6E-11	0.5945	2.0E-6	0.7472	2.3E-5	0.8513	7.8E-6	**0.8515**	4.9E-6
70%	0.8479	2.9E-10	0.5944	4.3E-6	0.7704	2.3E-5	0.8914	1.4E-5	**0.8915**	1.3E-5
80%	0.8984	8.7E-11	0.5963	6.6E-6	0.7942	8.4E-6	**0.9311**	3.6E-6	0.9306	4.8E-6
90%	0.9492	7.8E-11	0.5949	4.1E-6	0.8144	6.5E-6	**0.9653**	4.2E-6	**0.9653**	3.1E-6

Supplementary Table 2: The average and variance of AUPR scores of all competing prior-based algorithms. The best scores for prior networks with different percentage of true edges are in bold.

	Prior		MERLIN_P		Inferelator		NetREX($\kappa = 0$)		NetREX($\kappa = 1$)	
True edges %	AUPR	Var.	AUPR	Var.	AUPR	Var.	AUPR	Var.	AUPR	Var.
10%	0.0258	2.1E-8	0.0780	1.4E-7	0.0380	1.2E-4	0.0426	6.6E-5	**0.0432**	8.0E-5
20%	0.0577	5.2E-7	0.0775	1.6E-7	0.0962	1.6E-4	**0.1292**	1.9E-4	**0.1292**	1.6E-4
30%	0.1091	1.3E-6	0.0777	4.78E-7	0.1626	3.6E-4	**0.2285**	1.3E-4	**0.2285**	1.4E-4
40%	0.1785	4.5E-7	0.0783	5.6E-7	0.2293	3.3E-4	0.3305	3.8E-4	**0.3318**	3.4E-4
50%	0.2682	3.2E-6	0.0776	1.8E-7	0.2910	1.5E-4	0.4347	8.4E-5	**0.4357**	7.0E-5
60%	0.3751	4.1E-6	0.0769	3.8E-7	0.3573	1.7E-4	**0.5393**	7.0E-5	0.5383	6.7E-5
70%	0.5046	8.4E-6	0.0774	2.9E-7	0.4078	1.0E-4	0.6314	2.2E-4	**0.6326**	2.4E-4
80%	0.6493	4.7E-6	0.0770	6.1E-7	0.4689	2.6E-5	0.7271	7.6E-5	**0.7277**	9.4E-5
90%	0.8154	4.8E-6	0.0775	4.0E-7	0.5189	4.6E-5	**0.8230**	5.5E-5	0.8224	3.4E-5

Supplementary Table 3: The comparison of AUROC score for different ratios of true to false edges in the prior networks.

	Prior		Inferelator		NetREX		MERLIN_P	
True to false ratio	AUROC	Var.	AUROC	Var.	AUROC	Var.	AUROC	Var.
0:1	0.4966	3.1E-33	0.4948	7.7E-5	0.4977	5.8E-7	**0.5955**	1.3E-6
1:0	0.75	0	0.7603	2.1E-5	**0.8158**	8.8E-6	0.5941	4.7E-6
1:2	0.7431	6.0E-11	0.7030	6.1E-5	**0.7591**	6.0E-6	0.5961	5.9E-6
1:5	0.7328	1.1E-9	0.6627	4.6E-5	**0.7531**	1.4E-5	0.5971	5.6E-7
1:10	0.7156	1.1E-8	0.6295	6.2E-5	**0.7379**	2.5E-5	0.5956	1.2E-6

Supplementary Table 4: The comparison of AUPR score for different ratios of true to false edges in the prior networks.

	Prior		Inferelator		NetREX		MERLIN_P	
True to false ratio	AUPR	Var.	AUPR	Var.	AUPR	Var.	AUPR	Var.
0:1	0.0132	0	0.0131	4.7E-8	0.0133	1.9E-8	**0.0787**	6.9E-8
1:0	0.5201	0	0.4477	1.3E-4	**0.5610**	9.7E-5	0.0773	1.5E-6
1:2	0.1839	6.0E-7	0.2640	3.3E-4	**0.3203**	1.1E-4	0.0784	9.3E-8
1:5	0.0981	3.4E-7	0.1846	7.7E-5	**0.2241**	5.5E-5	0.0773	9.0E-7
1:10	0.0579	1.7E-7	0.1222	2.5E-4	**0.1544**	2.0E-4	0.0779	9.0E-9

Supplementary Table 5: The comparison of the methods based on the ability to identify novel interactions that were not used in the DREAM5 challenge as a function of quality of the prior network where the prior quality is measured as the percentage of true edges.

true edge	Inferelator			NetREX			MERLIN_P		
percentage	# TP	# FP	# Unique	# TP	# FP	# Unique	# TP	# FP	# Unique
10%	5 ± 2.82	15,094 ± 203.5	9	4 ± 2.97	3,710 ±80.4	8	24 ± 2.89	49,033 ±166.6	28
20%	9 ± 3.13	14,606 ± 337.4	14	5 ± 2.26	3,422 ± 80.7	21	23 ± 1.53	48,762 ± 325.8	27
30%	8 ± 2.02	14,480 ± 277.5	19	7 ± 1.90	3,078 ± 51.1	22	25 ± 0.58	49,066 ± 302.1	26
40%	10 ± 1.32	14,326 ± 270.0	18	10 ± 3.51	2,860 ± 96.5	26	25 ± 1.73	48,883 ± 420.0	28
50%	11 ± 3.07	14,227 ± 210.7	17	11 ± 2.25	2,613 ± 55.1	25	23 ± 2.08	48,648 ± 137.5	23
60%	13 ± 3.40	14,187 ± 348.4	19	14 ± 2.45	2,389 ± 64.7	25	25 ± 1.53	48,733 ± 58.9	28
70%	14 ± 2.28	13,948 ± 190.7	18	19 ± 1.78	2,256 ± 63.4	29	23 ± 1.15	48,417 ± 58.9	26
80%	13 ± 1.06	14,063 ± 249.4	19	20 ± 3.02	2,151 ± 63.4	33	25 ± 1.15	48,546 ± 573.5	28
90%	15 ± 1.58	13,833 ± 177.3	18	21 ± 2.64	1,994 ± 59.5	30	25 ± 1.25	48,446 ± 387.6	28

Elements in the supplementary table are means ± standard deviation. TF and FP are True Positives and False Positives.

Unique is the number of identified unique novel edges over 10 runs of the methods starting with randomly selected priors.

Supplementary Table 6: The comparison of the methods based on the ability to identify novel interactions that were not used in the DREAM5 challenge as a function of quality of the prior network where the prior quality is measured as the ratio of true to false edges.

ratio of	Inferelator			NetREX			MERLIN_P		
true to false	# TP	# FP	# Unique	# TP	# FP	# Unique	# TP	# FP	# Unique
1:0	16 ± 2.50	15,052 ± 124.8	25	19 ± 1.91	3,014 ± 56.4	26	25 ±1.91	46,493 ± 481.5	28
1:2	12 ± 4.67	15,085 ± 441.3	20	11 ± 2.36	5,088 ± 78.3	34	24 ± 0.00	47,033 ± 413.1	26
1:5	9 ± 4.48	15,292 ± 269.6	18	11 ± 2.27	8,187 ± 85.4	49	23 ± 0.58	46,751 ± 279.9	24
1:10	8 ± 3.21	15,252 ± 378.3	12	12 ± 3.41	13,363 ± 104.4	53	24 ± 0.58	47,187 ± 274.5	26
0:1	5 ± 4.81	15,454 ± 317.4	11	1 ± 1.96	3,032 ± 65.4	10	23 ± 2.08	46,607 ± 272.5	24

Elements in the supplementary table are means ± standard deviation. TF and FP are True Positives and False Positives.

Unique is the number of identified unique novel edges over 10 runs of the methods starting with randomly selected priors.

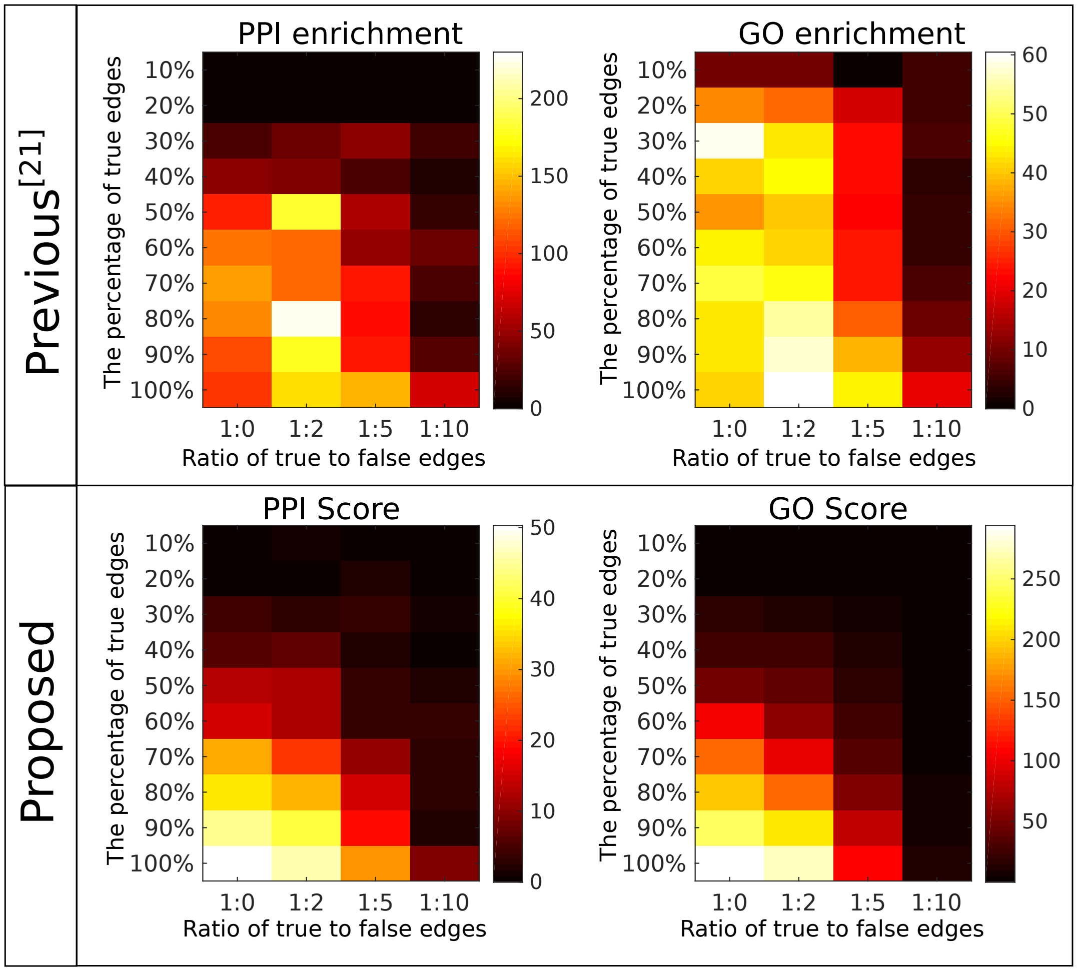

Supplementary Figure 6: Performance comparison of previous enrichment scores [24] and our proposed PPI and GO scores on *E.coli* GRNs with different noise levels. The simulated *E.coli* GRNs are generated based on the noise level, controlled by the percentage of the true edges and the ratio of true to false edges. Under each noise level, 10 randomly simulated *E.coli* GRNs are generated. PPI and GO scores are obtained by averaging over those 10 random networks. The first row contains the PPI and GO enrichment scores proposed in [24]. The second row includes the performance of our proposed PPI and GO scores. The comparison indicates that our proposed scores are more consistent with the quality of the networks than the previous ones [24]. Specifically, the higher the percentage of true edges and the lower the ratio of true to false edges, the higher the PPI and GO scores are.

Supplementary Table 7: Detailed prior networks information.

Prior networks for	# Genes	# TFs	# Interactions
Female	7,530	383	228,946
Male	8,529	363	203,068
Female without ovary genes	5916	383	156,066
Male without testis genes	6,698	347	156,436

We used the DREAM5 challenge *E. coli* dataset which has 807 samples and 2066 "gold standard" regulatory edges to explore sample size requirement. We used the "gold standard" *E. coli* network to generate a prior network that is estimated to have 20% of true edges (414 edges out of 2066 "gold standard" edges). The 20% threshold correspond to the threshold for which we start to observe improvements of the prediction made by NetREX over the prediction made by expression only methods. Then we applied NetREX to reconstruct the *E. coli* GRN given the same prior network and randomly selected expression data of various sizes. Specifically, each sample size we randomly selected 10 sets of samples and run NetREX. As shown in Fig. 7, when the sample size is less than 100, the performance of NetREX was quickly improving with the number of samples. After sample size reached ˜100, adding additional samples did not have a drastic effect. Interestingly, even with a small number of samples, NetREX provided an improvement over the prior network in terms of AUPR score.

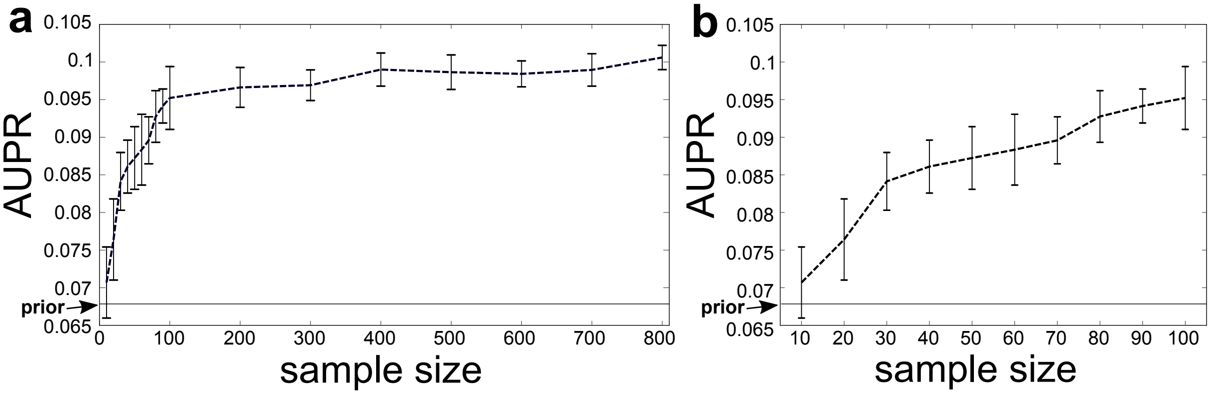

Supplementary Figure 7: Impact of sample size on NetREX performance. (a) The average and standard deviation of AUPR for different sample sizes. (b) The zoom in of (a) between sample size 10 and 100.

Supplementary Note 4. Results on *Drosophila* Data

Next we applied NetREX to gene expression data in adult female and male flies from 99 hemizygotic lines (deletion/+) of the *Drosophila* deletion collection (DrosDel) project covering 68% of chromosome 2L. Specifically, in each of the DrosDel lines a different chromosomal fragment has been deleted, leaving the organism with only one copy of genes for the deleted region [25]. We used the network in [24], which was constructed through integrating diverse functional genomics datasets (such as TF binding and evolutionarily conserved sequence motifs) in a supervised learn-

ing framework, as the prior network for NetREX . The data used in [24] generally came from cell lines and expression profiles of certain developmental stages. NetREX predicted regulatory networks for adult female flies, and we subsequently verified these networks using GO functional annotations [26], physical protein-protein interactions (PPIs), and target genes of the *Drosophila* TF doublesex (DSX) [27].

In order to reconstruct different sex-specific networks, the prior networks were also different. The detailed information of the 3 different prior networks used is listed in Supplementary Table 7.

Supplementary Table 8: Detailed information used for PPI and GO scores comparison for predicted *Drosophila* female-specific networks.

	# Co-regulated gene pairs	#PPIs	#Gene pairs (GO term similarity >=0.5)	PPI score	GO score
Prior	316670	659	972	74.113	3.9966
Genie3(top 50,000)	30528	64	301	229.6467	300.653
Genie3(top 100,000)	12798	49	139	153.6647	243.8724
Genie3(top 150,000)	7228	47	89	137.3332	129.4579
Genie3(top 200,000)	4951	48	61	138.2621	122.2693
Genie3(top 250,000)	3855	50	45	143.224	94.2473
Genie3(top 300,000)	3133	43	40	137.4621	80.0387
NetREX(top 50,000)	19737	111	157	**>324.698**	**>324.698**
NetREX(top 100,000)	43164	108	409	**>324.698**	**>324.698**
NetREX(top 150,000)	69048	112	448	**>324.698**	**>324.698**
NetREX(top 200,000)	75838	107	508	**>324.698**	**>324.698**
NetREX(top 250,000)	74857	108	525	302.1078	**>324.698**
NetREX(top 300,000)	74710	109	542	273.5249	**>324.698**

Detailed information for computing PPI and GO scores for the predicted female-specific, male-specific, and male-specific without genes highly expressed in the testis networks is listed in Supplementary Table 8, Supplementary Table 9, and Supplementary Table 10, respectively. For Genie3 and NetREX, because certain cutoffs needs to be selected in order to have a fair comparison, we show PPI and GO scores under different cutoffs. We found that networks inferred by NetREX achieve the best PPI and GO scores for female flies and networks predicted by Genie3 achieve the best PPI and GO scores for male files. After removing genes highly expressed in the testis from the prior network and the expression data, we found that NetREX performs better than Genie3.

Despite having used the same base prior network to reconstruct the sex-specific *Drosophila* GRNs, the quality of the predicted networks ended up being quite different. Based on the above evaluation, we found the prior network proposed in [24] to be female biased. In this section, we aim to assess the quality of the prior networks.

For female flies, $Q(G_0) = 6.4$ when $C = \{60,000,\ 80,000,\ 100,000,\ 120,000,\ 140,000\}$. For male flies, $Q(G_0) = -14.4$ when $C = \{60,000,\ 80,000,\ 100,000,\ 120,000,\ 140,000\}$. And for male flies where we remove genes highly expressed in the testis, $Q(G_0) = 4.1$ when $C = \{20,000,\ 40,000,\ \ 60,000,\ 80,000,\ 100,000\}$. The quality scores are consistent with the performance in terms of PPI and GO scores.

Supplementary Table 9: Detailed information used for PPI and GO scores comparison for predicted *Drosophila* male-specific networks.

	# Co-regulated gene pairs	#PPIs	#Gene pairs (GO term similarity >=0.5)	PPI score	GO score
Prior	273801	588	766	108.112	2.7996
Genie3(top 50,000)	114221	174	1321	**59.4729**	274.0408
Genie3(top 100,000)	80969	108	1187	28.1434	**>324.698**
Genie3(top 150,000)	63931	91	1166	27.6026	**>324.698**
Genie3(top 200,000)	53500	99	1131	46.4055	**>324.698**
Genie3(top 250,000)	46832	85	1129	40.1093	**>324.698**
Genie3(top 300,000)	39723	82	1009	44.5129	**>324.698**
NetREX(top 50,000)	17456	57	940	49.4762	**>324.698**
NetREX(top 100,000)	20712	34	538	21.4606	308.1195
NetREX(top 150,000)	27018	33	557	16.0788	220.8253
NetREX(top 200,000)	42193	34	458	9.358	185.5866
NetREX(top 250,000)	50242	42	502	10.0698	209.5641
NetREX(top 300,000)	68532	97	627	27.9719	151.8892

Supplementary Table 10: Detailed information used for PPI and GO scores comparison for predicted *Drosophila* male-specific networks without genes highly expressed in testis.

	# Co-regulated gene pairs	#PPIs	#Gene pairs (GO term similarity >=0.5)	PPI score	GO score
Prior	185710	342	584	53.3471	4.7076
Genie3(top 50,000)	30528	64	301	37.5303	71.6957
Genie3(top 100,000)	12798	49	139	44.6857	26.9861
Genie3(top 150,000)	7228	47	89	60.6501	21.1984
Genie3(top 200,000)	4951	48	61	75.5626	15.0099
Genie3(top 250,000)	3855	50	45	88.694	10.6838
NetREX(top 50,000)	47642	140	899	**137.77**	**324.698**
NetREX(top 100,000)	27826	90	605	110.27	251.53
NetREX(top 150,000)	28515	122	568	133.08	218.26
NetREX(top 200,000)	62551	185	644	83.23	109.86
NetREX(top 250,000)	88763	259	725	99.02	79.6111

Supplementary Table 11: DSX agreement on male-specific networks without genes highly expressed in testis.

	# predicted	# verfied	percentage	p-value
Prior	2	2	100%	3.10E-01
MERLIN_P	85	49	57.6%	4.30E-01
Inferelator	4	3	75%	4.10E-01
Genie3(Top 20 TFs)	145	103	71.03%	1.29E-04
Genie3(Top 30 TFs)	274	190	69.34%	2.90E-06
Genie3(Top 40 TFs)	427	285	66.74%	3.48E-06
NetREX(Top 20 TFs)	23	19	82.61%	7.21E-03
NetREX(Top 30 TFs)	46	37	80.43%	4.52E-04
NetREX(Top 40 TFs)	62	51	82.26%	1.15E-05

Background: 6698 genes in male-specific networks without genes highly expressed in testis and 3755 of them are DSX targets.

p-values are computed by hypergeometric test.

As several lines of evidence indicate that the organizational principles of the regulatory program of the testis is unique [28, 29, 30, 31, 32, 33, 34, 35, 36, 37, 38, 39]. The *Drosophila* testis has a radically different gene expression machinery compared to any other tissue [28, 29, 31]. There are probably several causes of this special gene expression profile. First, the testis expresses specific paralogous components of the basal transcriptional machinery (distinct TATA binding protein Associated Factors, or TAFs) [34]. Perhaps because of this specialized basal core promoter machinery, at least some spermatocyte gene expression requires only very short promoters, such as the 14 bases required for expression of a tubulin encoding gene in the testis [40]. Additionally, gene expression in the male germline is regulated more hierarchically, with high levels of expression in primary spermatocytes and then only low-level expression during meiosis and sperm maturation. This transcriptional control is mediated by testis-specific basal transcriptional machinery and core promoter sequences, not typical transcription factors and enhancers [36, 33, 34, 38, 39]. The bulk of regulation appears to occur by translational control [41, 42] and is thus not modeled by a transcription factor based network.

To further validate the predictions obtained from different methods, we concentrated on target genes of the *Drosophila* transcription factor doublesex (DSX) which is involved in the sex determination system as two different isoforms [43]. Recently, Clough et al. [27] reported a rich set of DSX targets based on a series of genome-wide experiments and analysis. According to [27], we treated a gene as a DSX target based on either its ChIP-Seq gene occupancy or its conserved motif scores ($IC > 2$). Thus, we checked how well the predicted DSX targets of NetREX are in agreement with genes identified in [27].

When we keep the genes highly expressed in ovary in our reconstructed female specific GRN, the DSX targets predicted by NetREX are highly overlapped with those reported in [27]. The details are shown in Supplementary Fig. 8. The results for female networks without genes highly expressed in ovary are shown in the main text (Fig.4 a, b, and c). We found NetREX significantly outperforms other methods in female flies with or without genes highly expressed in ovary. But

the GRN without genes highly expressed in ovary has better performance than the one with genes highly expressed in ovary.

a Predicted DSX targets supported by biological evidence

	DSX targets (Female): Computationally predicted	DSX targets (Female): Independently supported	Hypergeometric test: p-value
Prior	3	3	2.18E-01
MERLIN+P	47	34	5.73E-02
Inferelator	3	3	2.18E-01
NetREX(Top 20 TFs)	1900	1240	**8.52E-08**
NetREX(Top 30 TFs)	2451	1545	**2.41E-04**
NetREX(Top 40 TFs)	2922	1797	**3.36E-02**
Genie3(Top 20 TFs)	411	238	8.47E-01
Genie3(Top 30 TFs)	618	363	7.91E-01
Genie3(Top 40 TFs)	868	500	9.54E-01

Background: 7535 genes in female GRN inculding genes highly expressed in ovary and 4535 of them are DSX targets. p-values are computed by hypergeometric test.

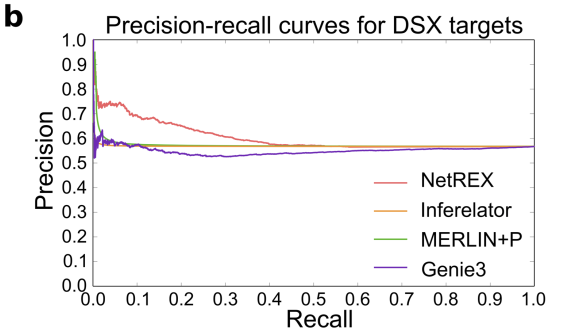

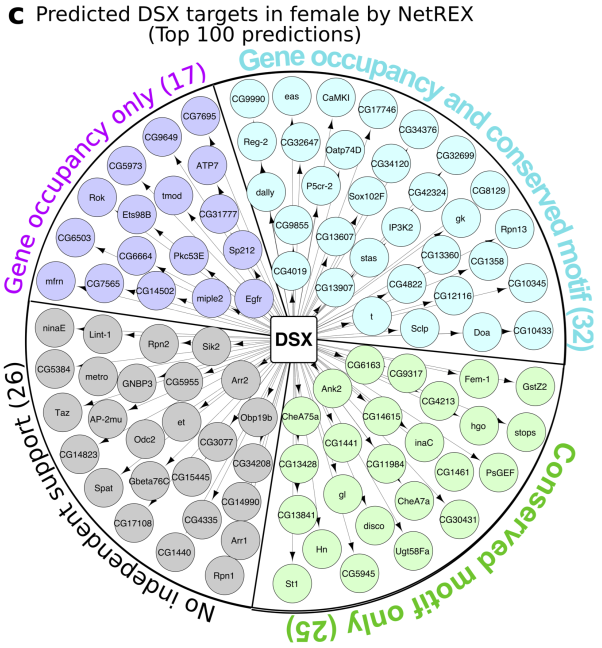

Supplementary Figure 8: Validation of predicted DXS targets (predicted with genes highly expressed in ovary). (a) Enrichment of experimentally supported DSX targets recovered by different methods for female GRN. Enrichment for male GRN without testis is shown in Supplementary Table 12. (b) Precision-recall curves for predicting DSX targets for compared methods. The DSX targets predicted by each method are ranked by assigned weights. A high area under the curve corresponds to high precision (low false positive rate) and high recall (low false negative rate). As the ground truth we use DSX targets reported in 8 based on ChiP-Seq occupancy and conserved motif scores. (c) Top 100 targets predicted by NetREX in the female GRN.

The result for the agreement in the male network without genes highly expressed in the testis is shown in Supplementary Table 11. After removing genes highly expressed in the testis, the performance of predicting DSX targets by NetREX is competitive to that of Genie3 in male flies.

We identified genes that are differentially expressed in male and female flies using independent sexed-tissue expression data obtained from GSE99574. We used expression data from only *Drosophila melanogaster* tissues (96 samples from GSM2647254 to GSM2647349) to identify sex differentially expressed genes. Those 96 samples come from 8 tissues, namely, whole body, gonad, reproductive tract, abdomen carcass, digestive system, genitalia or terminalia, and thorax. For each tissue, we considered genes as having sex biased expression when the absolute log2 fold change is larger than 2 as well as the adjusted p-value is less than 1.0E-3 (log2 fold change and adjusted p-value were computed by DESeq2 [44]). Then we identified sex differentially expressed genes by finding the union among the sex differentially expressed genes identified in each tissue.

We examined whether DSX targets were enriched in those differentially expressed genes (identified by the method introduced in the the above paragraph) using the hypergeometric test. All compared methods return ranked lists of predictions. They might predict different number of regulators for each gene. To fairly compare those GRNs we take for each method the k-best predictions for each gene. We set $k = 20, 30, 40$ and show the results for female GRN with and without genes

highly expressed in ovary in Supplementary Fig. 9

a

Female GRN	Network cutoff (20 TFs / gene)				Network cutoff (30 TFs / gene)				Network cutoff (40 TFs / gene)			
	# DSX targets	# DiffExp genes	% DiffExp genes	p-value	# DSX targets	# DiffExp genes	% DiffExp genes	p-value	# DSX targets	# DiffExp genes	% DiffExp genes	p-value
**NetREX**	1900	926	***48.74%***	***2.53E-28***	2451	1156	***47.16%***	***1.71E-29***	2922	1331	***45.56%***	***1.5E-26***
**Inferelator**	3	0	00.00%	<1.0	3	0	00.00%	<1.0	3	0	00.00%	<1.0
**MERLIN+P**	46	20	43.48%	<0.20	46	20	43.48%	<0.20	46	20	43.48%	<0.20
**Genie3**	411	160	38.93%	<0.4	618	251	40.61%	8.0E-3	868	364	41.94%	6.2E-3

Background: 7530 genes in the female GRN and 2869 of them are differential expressed (DiffExp) genes. The percentage of DiffExp genes is 38.10%
p-value is obtained from hypergeometric test.
Best performaners is each comparison are in bold italic font.

b

Female GRN (no ovary)	Network cutoff (20 TFs / gene)				Network cutoff (30 TFs / gene)				Network cutoff (40 TFs / gene)			
	# DSX targets	# DiffExp genes	% DiffExp genes	p-value	# DSX targets	# DiffExp genes	% DiffExp genes	p-value	# DSX targets	# DiffExp genes	% DiffExp genes	p-value
**NetREX**	63	43	***68.28%***	***4.62E-04***	96	61	***63.54%***	***9.98E-04***	144	93	***64.58%***	***2.90E-05***
**Inferelator**	3	0	00.00%	<1.0	3	0	00.00%	<1.0	3	0	00.00%	<1.0
**MERLIN+P**	228	94	41.23%	<1.0	232	98	42.24%	<1.0	414	138	33.33%	<1.0
**Genie3**	330	132	40.00%	<1.0	502	208	43.43%	<1.0	708	310	43.79%	<1.0

Background: 5916 genes in the male GRN (no testis) and 2869 of them are differential expressed (DiffExp) genes. The percentage of DiffExp genes is 48.50%
p-value is obtained from hypergeometric test.
Best performaners is each comparison are in bold italic font.

Supplementary Figure 9: Comparison of DSX targets in GRNs with/without genes highly expressed in ovary enriched in differentially expressed genes with different cutoffs. (a) Comparison when considering genes highly expressed in ovary. (b)Comparison when not considering genes highly expressed in ovary.

For a fair comparison of the GRNs predicted by different methods, we compared everything using the same cutoff. For example, if the cutoff is 30 TFs per gene, it means that each gene at most could have 30 TFs based on the edge weights. The detailed comparison with more cutoffs for both male (without testis) and female is shown in Supplementary Fig. 10

Male GRN (no testis)	Network cutoff (20 TFs / gene)				Network cutoff (30 TFs / gene)				Network cutoff (40 TFs / gene)			
	# DSX targets	# DiffExp genes	% DiffExp genes	p-value	# DSX targets	# DiffExp genes	% DiffExp genes	p-value	# DSX targets	# DiffExp genes	% DiffExp genes	p-value
**NetREX**	23	15	65.22%	<0.8	46	31	67.39%	<0.8	62	46	74.19%	<0.7
**Inferelator**	3	0	00.00%	<0.85	3	0	00.00%	<0.85	3	0	00.00%	<0.85
**MERLIN+P**	80	60	75.00%	<0.4	82	62	75.61%	<0.4	82	62	75.61%	<0.4
**Genie3**	145	106	73.10%	<0.50	274	203	74.09%	<0.6	427	320	74.94%	<0.4

Background: 6698 genes in the male GRN (no testis) and 4969 of them are differential expressed (DiffExp) genes. The percentage of DiffExp genes is 74.18%
p-value is obtained from hypergeometric test.

Supplementary Figure 10: Comparison of DSX targets in GRNs without genes highly expressed in testis enriched in differentially expressed genes with different cutoffs.

Supplementary Note 5. Different Metrics

The p-value of the hypergeometric test is a metric used for evaluating the performance of different approaches. Suppose there are N biological "gold standard" samples within M possible samples, and a method selects m samples where n of them are "gold standard". The hypergeometric test can identify whether the "gold standard" samples are over-represented in the selected samples. The p-value is then the probability that more than n "gold standard" samples are identified in m samples. Therefore, the lower the probability is, the better the method is. We then use $-\log_{10}(\text{p-value})$ instead of the p-value. Due to float precision (IEEE-754 Floating Point), the smallest p-value could be 0.5×10^{-324}, and the largest $-\log_{10}(\text{p-value})$ is 324.698.

The F-measure is defined as

$$2 \times \frac{\text{Precision} \times \text{Recall}}{\text{Precision} + \text{Recall}}, \tag{40}$$

where

$$\text{Precision} = \frac{|\mathcal{E}^p \cap \mathcal{E}|}{|\mathcal{E}^p|}, \ \text{Recall} = \frac{|\mathcal{E}^p \cap \mathcal{E}|}{|\mathcal{E}|}. \tag{41}$$

$\mathcal{E}$ and $\mathcal{E}^p$ are edge sets of the underlying regulatory network $\mathcal{G}$ and the predicted regulatory network, respectively. F-measure ranges from 0 to 1, where 1 denotes that the underlying $\mathcal{G}$ is fully recovered and 0 means the opposite.

Here, we want to emphasis the difference between F-measure and AUROC and AUPR scores. The F-measure disregards the ranking of the edges in the predicted networks, and instead measures the overlap between the predicted networks and the "gold standard" networks. In contrast, AUROC and AUPR scores focus more on the ranking of the edges in the predicted networks.

Supplementary Note 6. Parameter Selection

For all prior-based methods, when constructing a GRN using a prior network with a partial "gold standard", we can try different parameters and find the optimal parameter set using the "gold standard" information. However, when there is no "gold standard" information available, NetREX can work in a consensus manner that ranks the edges in the network based on networks predicted from different parameters [17]. For other competing methods, we tried different parameters and used the ones that yielded the best scores of interest.

For simulated data, the parameters used to generate simulated data are $L = 60, N = 500, M = 100$. The density of the "gold standard" GRN is 0.1. The noise level in simulated expression data E is controlled by $\sigma = \{0, 0.1, 0.2, 0.3, 0.4, 0.5, 0.6, 0.7, 0.8, 0.9, 1.0\}$. The percentage of true edges in $\mathcal{G}_0$ is controlled by $\theta = \{0.1, 0.2, 0.3, 0.4, 0.5, 0.6, 0.7, 0.8, 0.9\}$.

There are seven parameters for the NetREX algorithm, which are λ, η, κ, ξ, μ, a and b. We applied a grid search to find the optimal parameter set. The settings are as follows. We set $\eta - \lambda \in [0.2, 5]$ with interval 0.2, $\eta + \lambda \in [1, 50]$ with interval 1, $\kappa \in [0.1, 0.5]$ with interval 0.1, $\xi = \{0.1, 1\}$, $\mu = \{0.1, 1\}$ and $a = b = \max_{i,j}(\text{abs } E(i, j))$. We used the same parameter set for NetREX_NP except $\lambda = 0$ and $\eta \in [1, 50]$ with interval 1. For the NetREX_ℓ_1 algorithm, we used exactly the same parameters as in the NetREX algorithm.

To test the potential of the competing algorithms, for a certain noise level, we first applied a grid search to all algorithms to find their optimal parameters on one simulated dataset based on F-measure. Then we used the optimal parameters for the other 50 simulated datasets under the same noise level. We compared the performance of different algorithms based on F-measures.

For *E.coli* data, we tested the competing algorithms by using prior networks with different noise levels controlled by the percentage of true edges or the ratio of true to false edges. For prior networks of a certain noise level, as in the treatment of the simulation data, we used a grid search to get the optimal parameters for one prior network based on AUPR score. Then we applied the optimal parameters to the other 10 prior networks generated at the same noise level. The AUROC, AUPR and the number of identified novel interactions that were not used in DREAM5 were reported.

For Inferelator, we set the number of bootstrap times at 5, which is the same as in NetREX, and obtained the optimal prior weight based on the above training procedure. For MERLIN_P, we obtained the optimal prior network weight also based on the above training procedure. For Genie3, we use the AUROC and AUPR scores reported on the DREAM5 challenge website.

With respect to *Drosophila* data, for running NetREX with the consensus strategy to construct the female-specific network, we set the parameters in a certain range to make sure the total number of edges in the predicted networks was around $250,000$ and the number of edges overlapping with the prior network was between $120,000$ to $200,000$. Specifically, we set the number of total edges to $250,000$ and the number of kept edges varied from $120,000$ to $200,000$ with interval $10,000$. We used the same total edge and kept edge parameters when building the male-specific network. For constructing the male-specific network without genes highly expressed in the testis, we set parameters to keep the total number of edges in the predicted networks around $200,000$ and the number of edges overlapping with the prior network between $80,000$ to $150,000$. Specifically, we set the number of total edges to $200,000$ and the number of kept edges varied from $80,000$ to $150,000$ with interval $10,000$.

For Inferelator and MERLIN_P, we used the parameters suggested in [21].The only parameter used for GENIE3 is K.[45] suggests two settings, $K = M - 1$ and $K = \sqrt{M}$. We compared the results of these two Ks and found $K = M - 1$ to be better than $K = \sqrt{M}$. Therefore, we used $K = M - 1$ in our comparisons. When a cutoff was needed to obtain the final GRN, we ranked the weights predicted by GENIE3 and did cutoffs based on rank.

Supplementary References

[1] Jérôme Bolte, Shoham Sabach, and Marc Teboulle. "Proximal alternating linearized minimization for nonconvex and nonsmooth problems". In: *Mathematical Programming* 146.1-2 (2014), pp. 459–494.

[2] Dd Lee and Hs Seung. "Algorithms for non-negative matrix factorization". In: *Advances in neural information processing systems* 1 (2001), pp. 556–562. arXiv: `0408058v1 [arXiv:cs]`.

[3] Julien Mairal, Francis Bach, Jean Ponce, and Guillermo Sapiro. "Online dictionary learning for sparse coding". In: *Proceedings of the 26th International Conference on Machine Learning* (2009), pp. 1–8. arXiv: `0908.0050`.

[4] Yu-Xiong Wang and Yu-Jin Zhang. "Nonnegative Matrix Factorization: A Comprehensive Review". In: *IEEE Transactions on Knowledge and Data Engineering* 25.6 (2013), pp. 1336–1353.

[5] James C Liao et al. "Network component analysis: reconstruction of regulatory signals in biological systems." In: *Proceedings of the National Academy of Sciences of the United States of America* 100.26 (2003), pp. 15522–15527.

[6] Linh M. Tran et al. "gNCA: A framework for determining transcription factor activity based on transcriptome: Identifiability and numerical implementation". In: *Metabolic Engineering* 7.2 (2005), pp. 128–141.

[7] Simon J. Galbraith, Linh M. Tran, and James C. Liao. "Transcriptome network component analysis with limited microarray data". In: *Bioinformatics* 22.15 (2006), pp. 1886–1894.

[8] Chunqi Chang, Zhi Ding, Yeung Sam Hung, and Peter Chin Wan Fung. "Fast network component analysis (FastNCA) for gene regulatory network reconstruction from microarray data". In: *Bioinformatics* 24.11 (2008), pp. 1349–1358.

[9] Neil Jacklin, Zhi Ding, Wei Chen, and Chunqi Chang. "Noniterative convex optimization methods for network component analysis". In: *IEEE/ACM Transactions on Computational Biology and Bioinformatics* 9.5 (2012), pp. 1472–1481.

[10] Amina Noor et al. "ROBNCA: Robust network component analysis for recovering transcription factor activities". In: *Proceedings - IEEE International Workshop on Genomic Signal Processing and Statistics* 29.19 (2013), pp. 19–22.

[11] Mikhail Belkin and Partha Niyogi. "Laplacian Eigenmaps for Dimensionality Reduction and Data Representation". In: *Neural Computation* 15.6 (2003), pp. 1373–1396. arXiv: `arXiv:1011.1669v3`.

[12] H Zou and T Hastie. "Regularization and variable selection via the elastic-net". In: *Journal of the Royal Statistical Society* 67 (2005), pp. 301–320.

[13] Nanne Aben, Daniel J. Vis, Magali Michaut, and Lodewyk F.a. Wessels. "TANDEM: a two-stage approach to maximize interpretability of drug response models based on multiple molecular data types". In: *Bioinformatics* 32.17 (2016), pp. i413–i420.

[14] Jishnu Das et al. "ENCAPP: elastic-net-based prognosis prediction and biomarker discovery for human cancers." In: *BMC genomics* 16.1 (2015), p. 263.

[15] L. Grippo and M. Sciandrone. "On the convergence of the block nonlinear Gauss-Seidel method under convex constraints". In: *Operations Research Letters* 26.3 (2000), pp. 127–136.

[16] Neal Parikh and Stephen Boyd. "Proximal Algorithms". In: *Foundations and Trends in Optimization* 1.3 (2013), pp. 123–231.

[17] Daniel Marbach et al. "Revealing strengths and weaknesses of methods for gene network inference." In: *Proceedings of the National Academy of Sciences of the United States of America* 107.14 (2010), pp. 6286–91.

[18] Daniel Marbach et al. "Wisdom of crowds for robust gene network inference". In: *Nature Methods* 9.8 (2012), pp. 796–804.

[19] D M Hamby. "A review of techniques for parameter sensitivity analysis of environmental models." In: *Environ. Monit. Assess.* 32.2 (1994), pp. 135–154.

[20] Shaogang Ren et al. "A Scalable Algorithm for Structured Kernel Feature Selection". In: *Proceedings of the Eighteenth International Conference on Artificial Intelligence and Statistics* 38 (2015), pp. 781–789.

[21] Alireza F Siahpirani and Sushmita Roy. "A prior-based integrative framework for functional transcriptional regulatory network inference." In: *Nucleic acids research* 45.4 (2016), gkw963.

[22] Mario L. Arrieta-Ortiz et al. "An experimentally supported model of the Bacillus subtilis global transcriptional regulatory network." In: *Molecular systems biology* 11.11 (2015), p. 839.

[23] Socorro Gama-Castro et al. "RegulonDB version 9.0: High-level integration of gene regulation, coexpression, motif clustering and beyond". In: *Nucleic Acids Research* 44.D1 (2016), pp. D133–D143.

[24] Daniel Marbach et al. "Predictive regulatory models in Drosophila melanogaster by integrative inference of transcriptional networks". In: *Genome Research* 22.7 (2012), pp. 1334–1349.

[25] Edward Ryder et al. "The DrosDel deletion collection: A Drosophila genomewide chromosomal deficiency resource". In: *Genetics* 177.1 (2007), pp. 615–629.

[26] J. a. Blake et al. "Gene ontology consortium: Going forward". In: *Nucleic Acids Research* 43.D1 (2015), pp. D1049–D1056.

[27] Emily Clough et al. "Sex- and tissue-specific functions of drosophila doublesex transcription factor target genes". In: *Developmental Cell* 31.6 (2014), pp. 761–773. arXiv: `NIHMS150003`.

[28] Brenton R Graveley et al. "The developmental transcriptome of Drosophila melanogaster." In: *Nature* 471.7339 (2011), pp. 473–9.

[29] Justen Andrews et al. "Gene Discovery Using Computational and Microarray Analysis of Transcription in the Drosophila melanogaster Testis". In: *Genome Research* 301 (2000), pp. 2030–2043.

[30] M Parisi et al. "Paucity of genes on the Drosophila X chromosome showing male-biased expression". In: *Science* 299.5607 (2003), pp. 697–700. arXiv: `NIHMS150003`.

[31] Michael Parisi et al. “A survey of ovary-, testis-, and soma-biased gene expression in Drosophila melanogaster adults.” In: *Genome biology* 5.6 (2004), R40.

[32] James B Brown et al. “Diversity and dynamics of the Drosophila transcriptome.” In: *Nature* 512.7515 (2014), pp. 1–7.

[33] Chenggang Lu and Margaret T. Fuller. “Recruitment of Mediator Complex by Cell Type and Stage-Specific Factors Required for Tissue-Specific TAF Dependent Gene Activation in an Adult Stem Cell Lineage”. In: *PLoS Genetics* 11.12 (2015), pp. 1–24.

[34] Mark Hiller et al. “Testis-specific TAF homologs collaborate to control a tissue-specific transcription program.” In: *Development (Cambridge, England)* 131 (2004), pp. 5297–5308.

[35] X. Chen. “Tissue-Specific TAFs Counteract Polycomb to Turn on Terminal Differentiation”. In: *Science* 310.5749 (2005), pp. 869–872.

[36] a Santel, J Kaufmann, R Hyland, and R Renkawitz-Pohl. “The initiator element of the Drosophila beta2 tubulin gene core promoter contributes to gene expression in vivo but is not required for male germ-cell specific expression.” In: *Nucleic acids research* 28.6 (2000), pp. 1439–1446.

[37] Beata Bielinska, Jining Lü, David Sturgill, and Brian Oliver. “Core promoter sequences contribute to ovo-B regulation in the Drosophila melanogaster germline”. In: *Genetics* 169.1 (2005), pp. 161–172.

[38] Oxana M. Olenkina et al. “Promoter contribution to the testis-specific expression of Stellate gene family in Drosophila melanogaster”. In: *Gene* 499.1 (2012), pp. 143–153.

[39] Yongsheng Bai, Claudio Casola, and Esther Betrán. “Quality of regulatory elements in Drosophila retrogenes”. In: *Genomics* 93.1 (2009), pp. 83–89.

[40] F Michiels, a Gasch, B Kaltschmidt, and R Renkawitz-Pohl. “A 14 bp promoter element directs the testis specificity of the Drosophila beta 2 tubulin gene.” In: *The EMBO journal* 8.5 (1989), pp. 1559–1565.

[41] E Kempe, B Muhs, and M Schäfer. “Gene regulation in Drosophila spermatogenesis: analysis of protein binding at the translational control element TCE.” In: *Dev. Genet.* 14 (1993), pp. 449–459.

[42] Rebeccah J. Katzenberger et al. “The Drosophila Translational Control Element (TCE) Is Required for High-Level Transcription of Many Genes That Are Specifically Expressed in Testes”. In: *PLoS ONE* 7.9 (2012).

[43] B S Baker. “Sex in flies: the splice of life.” In: *Nature* 340.6234 (1989), pp. 521–524.

[44] Michael I. Love, Wolfgang Huber, and Simon Anders. “Moderated estimation of fold change and dispersion for RNA-seq data with DESeq2”. In: *Genome Biology* 15.12 (2014), pp. 1–21.

[45] Vân Anh Huynh-Thu, Alexandre Irrthum, Louis Wehenkel, and Pierre Geurts. “Inferring regulatory networks from expression data using tree-based methods”. In: *PLoS ONE* 5.9 (2010), pp. 1–10.
