# Supplementary material for: Reprogramming of regulatory network using expression uncovers sex-specific gene regulation in Drosophila
Source: Nat Commun. 2018 Oct 3;9:4061. doi: 10.1038/s41467-018-06382-z (PMC6170494; doi:10.1038/s41467-018-06382-z)
Supplement: Supplementary file 3 — Description of Additional Supplementary Files [file 41467_2018_6382_MOESM3_ESM.pdf]

Description of Additional Supplementary Files

File Name: Supplementary Data 1
Description: Predicted female-specific GRN

File Name: Supplementary Data 2
Description: Predicted male-specific GRN
